# Beyond Equilibrium: Tracing Redox-Driven Pathways That Shape Aqueous and Mineral Chemistry

**DOI:** 10.1021/acs.est.6c08480

**Published:** 2026-10-14

**Authors:** Shishir Mundra, Mohit Pundir, Barbara Lothenbach, David S. Kammer, Ueli M. Angst

**Affiliations:** 1 Institute for Building Materials (IfB), 27219 ETH Zurich, Laura-Hezner-Weg 7, 8093 Zurich, Switzerland; 2 EMPA, Laboratory for Concrete and Asphalt, CH-8600 Dübendorf, Switzerland

**Keywords:** Redox reactions, Reactive-transport framework, Oxidation states, Thermodynamic speciation, Kinetics

## Abstract

Numerous aqueous and geochemical systems host elements in multiple redox states, with wide ranging implications such as their influence on the formation/dissolution of minerals, water toxicity, and nutrient cycling. To uncover governing mechanisms and complex chemical interactions in geochemical systems, reactive-transport models have increasingly gained importance. However, their predictive capabilities remain limited because existing approaches struggle to accurately account for the full complexities of redox reactions. Here, we develop a reactive-transport framework that leverages recent advances in thermodynamic modeling, speciation chemistry, and redox kinetics. Distinct from traditional models, we uniquely treat redox kinetics along with transport as transient phenomena, decoupled from Gibbs free energy minimization. By ensuring ionic concentrations are governed by nonequilibrium rate laws, this approach allows predicting the tempo-spatial distribution of speciation and precipitation of species across oxidation states. We illustrate the versatility of our framework through two case studies: manganese speciation in natural waters and the fate of dissolved iron in aqueous/porous media. Our framework significantly enhances the modeling of a diverse range of redox-sensitive environments.

## Graphical abstract

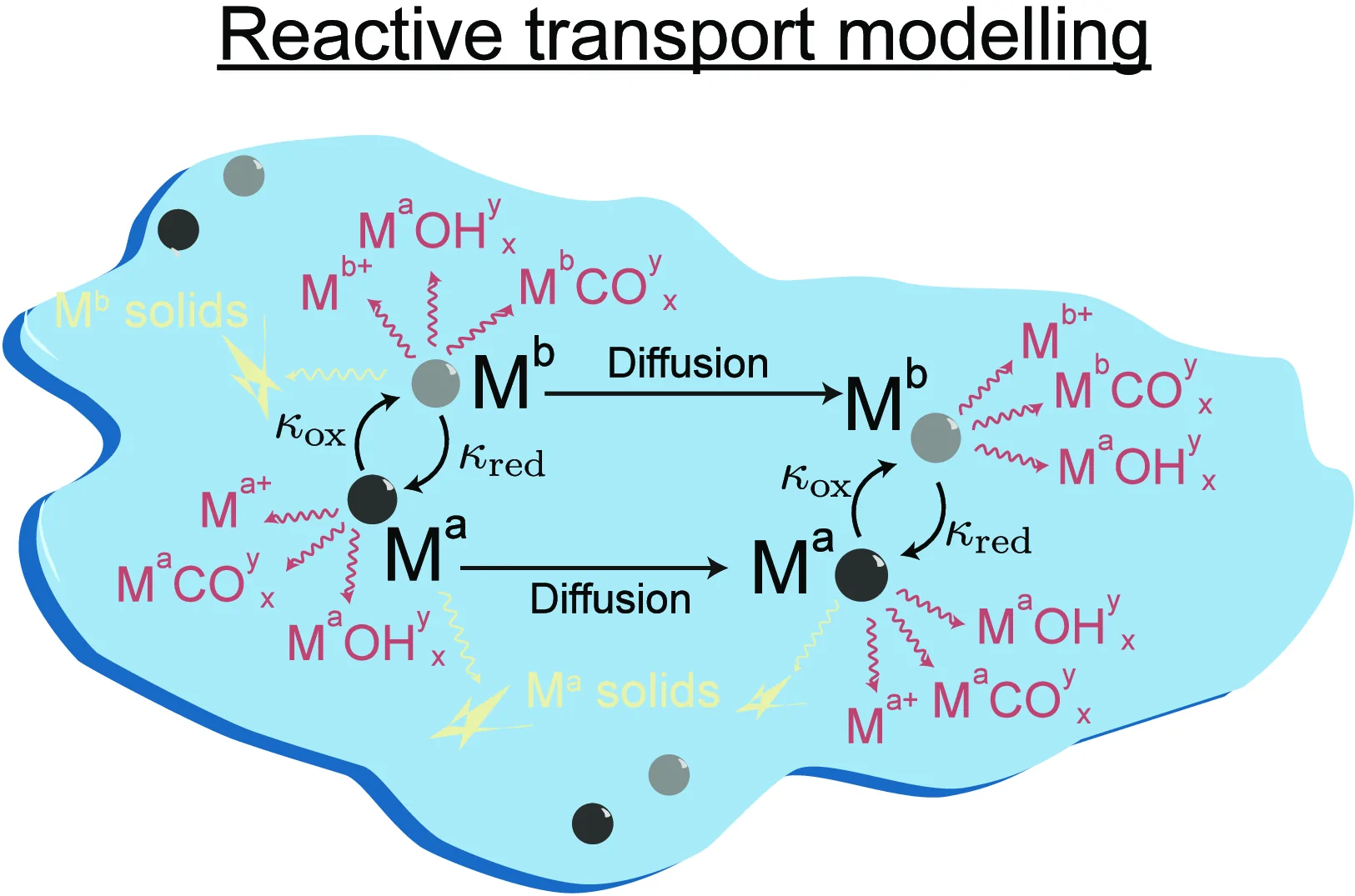

## 1 Introduction

Several elements (such as Fe, Mn, Cr) can coexist in multiple redox states in natural and engineered aqueous environments. The activity of an element in its various redox states influences both the thermodynamic state of the aqueous system and processes like the precipitation or dissolution of solid phases, as well as the transport of aqueous species.^1^ Across multiple oxidation states, the activity of an element is kinetically controlled by the local environmental conditions, such as pH, temperature, pO_2_, concentration of oxidants and reductants, complexation, adsorption or nucleation sites.^1^ For instance, the oxidation of dissolved Fe^II^ to Fe^III^ in different aqueous and porous environments proceeds through multiple simultaneous oxidation reactions depending on the pH and the prevalent Fe-species.^2–6^ Similar complex reaction schemes exist for other elements^7,8^ such as the oxidation and reduction reactions involving aqueous Mn^II^, Mn^III^ or Mn^IV^ in oceanic water^9–12^ and Cr^III^, Cr^IV^, Cr^V^ and Cr^VI^ in groundwater.^13,14^ To understand the impact of these elements’ oxidation states on their environment and its processes, it is essential to accurately predict their spatial and temporal evolution. Predicting such complex reactions, involving multiple species, kinetically controlled conversion, and transport, poses challenges and often necessitates advanced modeling techniques.^15,16^

Reactive transport models couple the reaction process with the transport process to simulate evolution of chemical species across space and time.^17^ Most reactive transport models solve the coupled equations using an operator split approach, where, in each iteration step, the conservative transport equations are solved first, followed by solving the chemical reaction terms using the transported concentrations.^18,19^ This method facilitates the use of advanced thermodynamic solvers like GEM-Selektor,^20^ Reaktoro,^21^ and PHREEQC^22^ to handle complex chemical reactions either in total equilibrium or in partial equilibrium for kinetically driven reactions.^23^ However, their usage necessitates accurate data sets on the equilibrium constants for each of the species involved in the chemical system.^23–25^ While this approach has been effective for various chemical environments^24,26^ including subsurface environments,^27–32^ it presents challenges, particularly when considering kinetically controlled multiple redox reactions. Oxidation and reduction reactions are constituted of several reaction pathways involving the speciation of elements in different oxidation states,^2,7–14^ each with a different kinetic constant. Solving such kinetically driven chemical systems in partial equilibrium requires accurate knowledge of the kinetic constants and the molar fractions of each of the species involved in the redox reaction and its pathways. However, numerical estimates of the kinetic rate constants for each redox reaction pathway deconvoluted from the experimentally derived rate law commonly reported, vary by several orders of magnitude.^4,5,33–36^ Reported oxidation kinetic rate constant (log *k*) values for the same Fe­(II) species vary by up to ∼ 5 orders of magnitude. For example, log *k* for Fe^2+^ ranges from −4.26 to +0.92 and for Fe­(OH)_2_ (aq),^4,5,33–36^ varies from ∼6.1 to 8.7, depending on the ionic strength.^4,5,33–36^ Even for hydrolyzed or carbonate complexes, interstudy differences of 1–3 orders of magnitude persist,^4,33–35^ quantitatively demonstrating substantial methodological and speciation-dependent discrepancies in the reported oxidation kinetics. Such large variations, compounded with incomplete thermodynamic datasets on aqueous speciation, has inhibited partial equilibrium solvers to accurately predict the fate of elements in different redox states and their consequences on dissolution or precipitation behavior.

To overcome these challenges associated with modeling of chemical systems that involve elements in multiple redox states, we introduce a novel reactive transport framework based on Gibbs free energy minimization. We employ a staggered approach, where we first consider kinetic rate laws governing redox reactions along with other transient phenomena such as transport. Unlike partial equilibrium approaches where the entire chemical system including all oxidation states is solved in a monolithic manner, here we decouple the Gibbs free energy minimization for deciphering the speciation and precipitation of elements in different oxidations states. We take advantage of the availability of accurate thermodynamic data sets to iteratively solve the decoupled systems. Furthermore, this decoupling enables the use of kinetic constants of overall redox reactions, for which reliable experimental data is available.^2,11,12^ This allows our reactive transport framework to simultaneously predict the fate of elements in multiple redox states (thermodynamically stable and metastable species) and its influence on the evolution of the overall chemistry of the aqueous system both in space and time.

## 2 Fundamental Concept behind the Proposed Reactive-Transport Framework

We propose a reactive-transport framework that incorporates the key mechanisms affecting a redox chemical system, such as hydrolysis and complexation, precipitation, kinetics of redox reactions, and the transport of aqueous and solid species (Figure 1a). We differentiate the above mechanisms as either transient (time-dependent) or equilibrium processes (indicated in blue and red, respectively in Figure 1e). A unique feature in our approach is that we consider the kinetically controlled oxidation and reduction of metal ions, along with the transport of all aqueous and solid species, as transient processes (Figure 1e). By considering the rate laws governing redox reactions as transient processes, thus separately from thermodynamic modeling, we ensure that the concentrations of metal ions in different oxidation states are determined by nonequilibrium processes^37^ (Figure 1b). Numerically, we implement this by introducing variables [M^a^] and [M^b^] for aqueous ions in each redox state, formed either because of oxidation or reduction. Here, [M^a^] and [M^b^] denote the sum of the concentration of all aqueous complexes of an element M in oxidation state *a* and *b*, respectively (Figure 1a, e). This approach enables us to use rate constants of oxidation (κ_ox_) or reduction (κ_red_), that can be experimentally determined and for which generally reliable data is available in the literature.^2,4,11^ Consequently, we tackle the challenges pertaining to the limited availability of kinetic rate constants for each of the species participating in redox reactions (Figure S1b). The spatial and temporal evolution of [M^a^] depends not only on its diffusion but also on its rate of conversion (oxidation or reduction) to [M^b^] (shown within blue regions in Figure 1e). Within each time increment, we first address the transient phenomena for each aqueous species, see (Section 5 for more details) and then utilize the acquired concentration values to address the equilibrium phenomena (red regions in Figure 1e).

**Figure 1:**
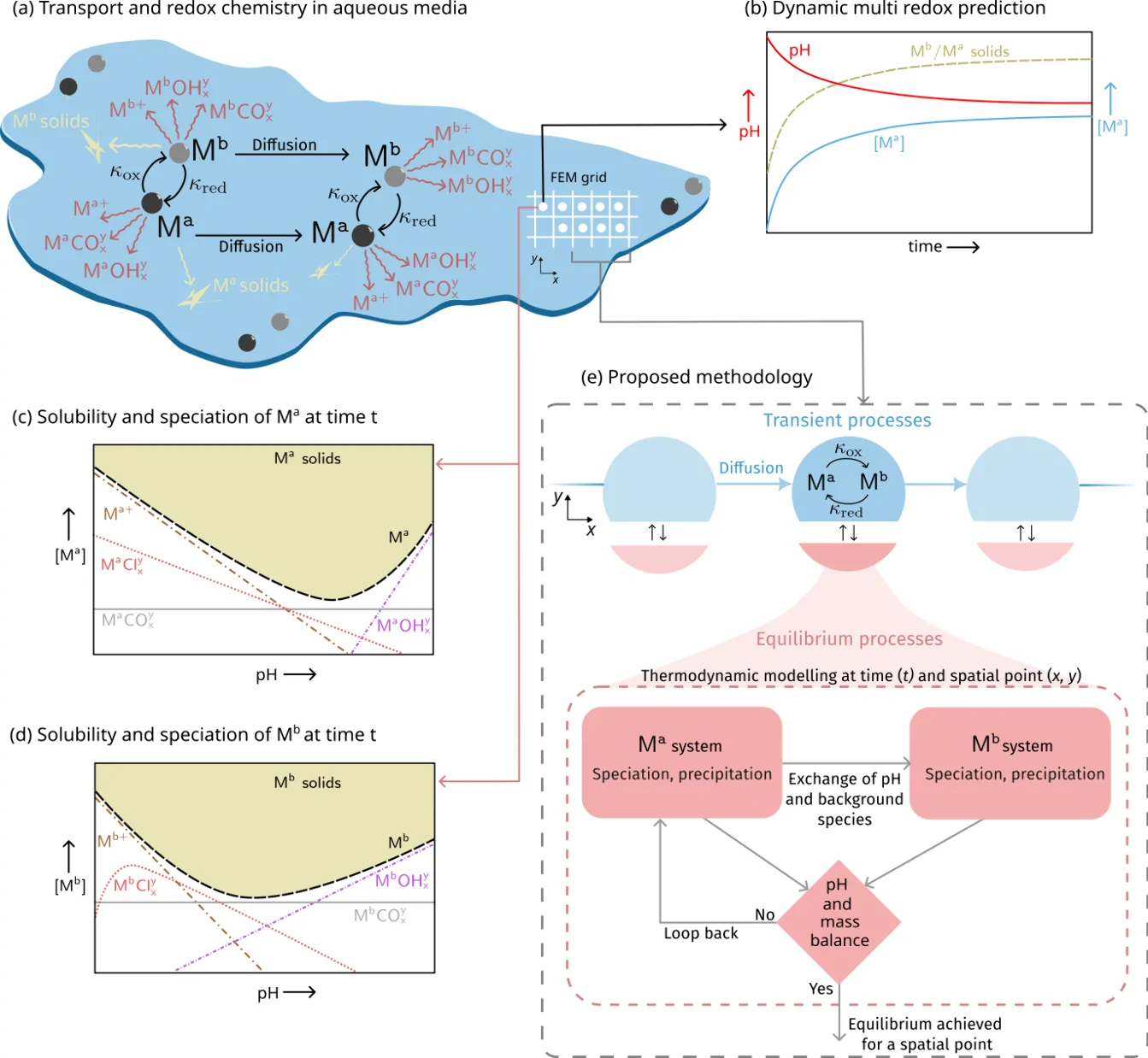
**Conceptual modeling approach for solving the spatial and temporal evolution of multiredox species in the proposed reactive transport framework:** (a) Schematic of the complex interplay between various kinetically controlled processes such as speciation, redox reactions, precipitation and transport, governing the fate of element (M) in multiple redox states (M^a^ and M^b^, where *a* > 0, *b* > *a*, and *a* and *b* are positive integers) in aqueous media. FEM stands for finite-element method. (b) The temporal evolution of multi-redox species (M^a^ and M^b^) at a certain location in space is governed by ion transport and kinetically controlled redox reactions that depend on electrolyte chemistry and (c, d) pH-dependent aqueous speciation and solubility of M^a^ and M^b^. (e) Our proposed methodology navigates such complexities of redox sensitive aqueous environments by decoupling transient (blue) and equilibrium processes (red). The proposed method solves for hydrolysis, speciation, oxidation or reduction, precipitation, dissolution, transport of elements (in multiple redox states).

After solving the transient processes, we utilize the variables [M^a^] and [M^b^] to solve for the speciation and precipitation of ions in multiple redox state at every spatial point (Figure 1c, d). Here, we separate speciation and precipitation of one oxidation state from the speciation and precipitation of the other oxidation state (represented by red in Figure 1e). For this, we take the [M^a^] corresponding to oxidation state *a* at a spatial point, obtained after diffusion and oxidation/reduction from the first stage (indicated in blue in Figure 1e), along with the remaining amount of background species. We solve this chemical system under thermodynamic equilibrium (detailed in Section 5) under varying environmental conditions such as pH, partial pressures of CO_2_ and O_2_, and temperatures. Next, we consider a separate chemical system that comprises of [M^b^] corresponding to a different oxidation state, *b*, (obtained from the oxidation or reduction considered as transient processes) and the remaining background species (obtained from the previous chemical system). We solve the chemical systems corresponding to different oxidation states in parallel and in an iterative manner where all the background species are constantly exchanged between the systems, ensuring they converge to the same pH value (see Figure 1) and are mass balanced. The concentration of different species determined in this way (including multiple redox complexes, background species, and pH) reflects the concentration at the end of the time interval and serves as the starting point for the subsequent time step. Please refer to Section 5 for a detailed description of the methodology.

## 3 Application of the Reactive-Transport Framework

We showcase the applicability and capabilities of our reactive transport framework through two examples: Case I: the fate of electrochemically dissolved iron in porous media (Figure 2a), and, Case II: manganese speciation in natural waters (Figure 2b). Iron (Fe) and manganese (Mn) are among the most abundant transition metals on earth and are critical elements in various socio-economically important engineered aqueous and natural geochemical environments. Their redox chemistry not only influences several biogeochemical cycles but also plays an important role in the functioning of critical infrastructure systems. In the selected examples, both Mn and Fe can exist in multiple oxidation states over different length and time scales and influence a multitude of local environmental conditions and processes. Therefore, knowledge of the spatial and temporal distribution of different species is critical.

**Figure 2:**
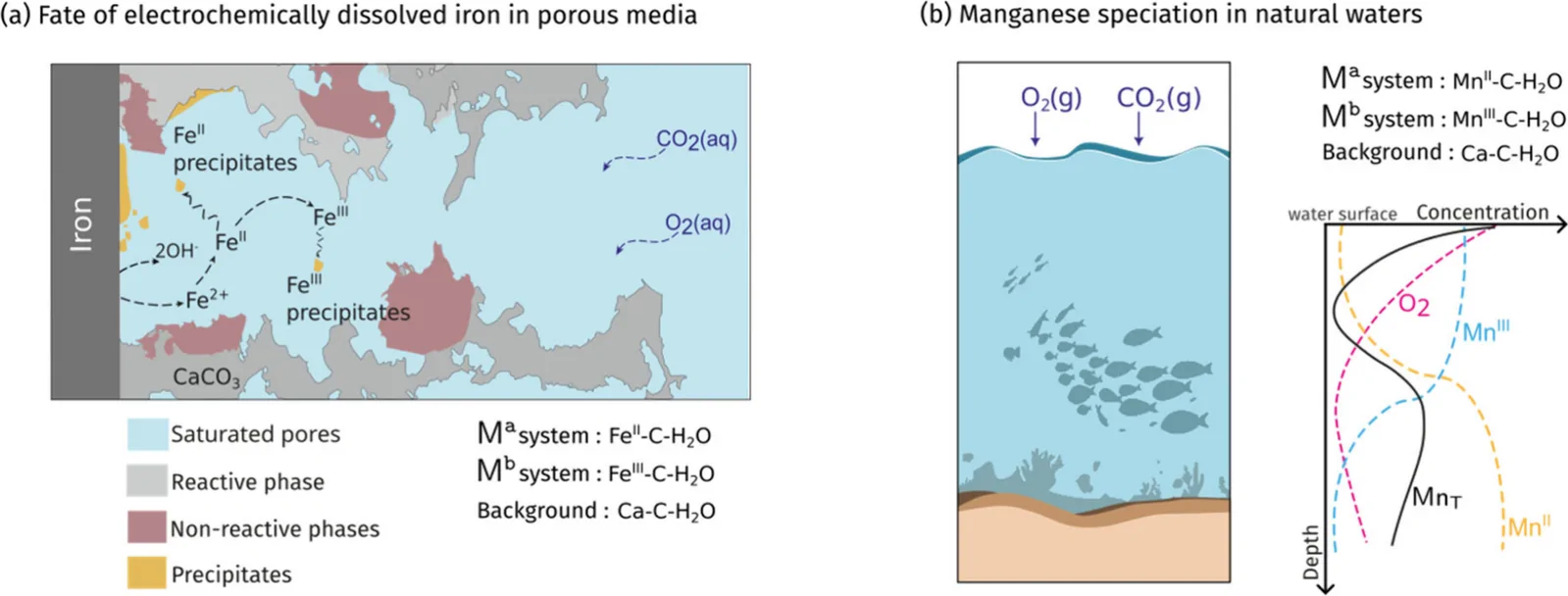
**Application examples of the proposed reactive transport framework**: (a) Case study I: On the fate of electrochemically dissolved iron in porous media. The interface between iron or steel and the porous medium is a multicomponent system comprising of reactive phases, CaCO_3_ (gray), nonreactive phases (brown), and aqueous phases such as pores saturated with moisture (blue). (b) Case study II: On the speciation of Mn in natural waters. Mn can exist in multiple oxidation states (Mn^II^, Mn^III^, Mn^IV^), and each of these species can play an important role in the biogeochemical cycles of several elements.

### 3.1 Case I: Electrochemically Dissolved Iron in Reactive Porous Media

The electrochemical dissolution of iron in porous media such as soils,^38–40^ concrete^41^ and other geological ecosystems,^42,43^ proceeds by the anodic release of Fe^2+^, and the simultaneous cathodic reduction of water or oxygen (depending on pO_2_), releasing OH^–^. Fe^2+^ can hydrolyze and form complexes with other anions, oxidize to Fe^III^ species, precipitate as a solid Fe^II^ phase, and diffuse away from the steel surface (Figure 2a). Simultaneously, Fe^III^ ions could precipitate as Fe^III^ solid phases, sorb onto solid surfaces, and also diffuse away from the metal surface (Figure 2a). These processes are highly interdependent and are in kinetic competition with each other. Their individual rates are determined by the prevailing chemical environment (pH, concentration of other ionic species, pO_2_) and are governed by the thermodynamic stability of the aqueous and solid phases. Importantly, each of these processes can dynamically alter the chemistry of the local environment (Supplementary Note 5), which thereby influences the rates of dissolution, oxidation, precipitation, sorption as well as shifts the thermodynamic equilibria.

Reactive transport models^39,44,45^ used to resolve the fate of iron in porous media, have two main limitations: a) they consider the rate of oxidation of Fe^II^ to Fe^III^ to be constant and not evolving as a function of the prevailing chemistry of the aqueous phase (despite the pronounced influence of e.g. pH^2,36^ ), and b) the reaction term cannot account for the thermodynamics of Fe^II^ and Fe^III^ aqueous and solid phases simultaneously. In our framework, considering Fe^II^ oxidation reactions within the transient processes allows us to decouple the Gibbs free energy minimization for metastable Fe^II^ and the more stable Fe^III^ species. Ensuring the composition of the background species (in a porous medium) within each of the minimization schemes is the same for Fe^II^ and Fe^III^ species, we accurately predict the activity of the Fe^II^ and Fe^III^ species at every time step. This allows us to consider the kinetic rate constant for Fe^II^ oxidation as a function of the activity and speciation of Fe^II^, and other parameters such as pH and pO_2_.

To illustrate this, we here employ the example of iron or steel dissolution in porous media,^38–41^ a longstanding challenge with a large socio-economic impact.^46^ For instance, consider corrosion in a porous medium such as steel in carbonated concrete fully saturated with an aqueous electrolyte (Figure 2a and Supplementary Note 5) or corrosion of an archeological artifact or steel pipes in soil (with the reactive soil particles here simplified as CaCO_3_). We apply our proposed framework to understand the influence of three key parameters, (a) porosity of the porous medium, (b) the concentration of reactive solid phases such as CaCO_3_ surrounding the pores and (c) the presence or absence of oxygen, on the chemical evolution of the metal-porous medium interface as well as of the surrounding matrix.

Our simulations (Section 5.4) suggest that the pH at the metal-porous medium interface is not constant and evolves over time depending on the porosity (Figure 3a), the amount of reactive solid phases such as CaCO_3_ surrounding the pores (Figure 3e), and the presence (Figure 3i) or absence (Figure 3l) of dissolved oxygen (Supplementary Note 5). The interfacial pH decreases with time (Figure 3a, e, i, l), which can be attributed to various pH dependent processes such as oxidation of Fe^II^ ions,^2–6^ hydrolysis and speciation of Fe^II^ and Fe^III^ ions,^47^ and the precipitation of thermodynamically stable Fe^II^ or Fe^III^ solid phases^47,48^ (see Supplementary Note 5). As the interfacial pH decreases, the solubility of Fe^II^ increases^47^ and therefore, the concentration of Fe^II^ species at the interface increases (Figure 3b, f, j, m). However, as the concentration of Fe^II^ is also dynamically controlled by the pH and pO_2_ dependent oxidation rate of Fe^II^ (or source of Fe^III^), the concentration of Fe^II^ does not necessarily follow the solubility limit of Fe^II^ with respect to Fe­(OH)_2_ (s) or FeCO_3_ (s) (Supplementary Note 5.3), which is a feature traditional modeling frameworks struggle accounting for.^23,24^ We observe this when comparing Figure 3j and Figure 3m, where the [Fe^II^] at the metal-porous medium interface is much higher in anaerobic environments than aerobic environments, even though the pH is lower in the case of the latter. Furthermore, our framework allows untangling the aqueous speciation of Fe^II^ and Fe^III^ temporally and spatially, and assess the concentration of each of the species participating in the oxidation reactions. The majority of the interfacial Fe^II^ initially exists predominantly in the form of FeCO_3_ (aq) and Fe^2+^ (Figure 3c and Figure 3g). Hydrolysis products of Fe^2+^ such as FeOH^+^, Fe­(OH)_2_ (aq), and Fe­(OH)_3_^–^ are only present in trace quantities at the pH of the interface. Upon time and a decrease in the pH, nonhydrolyzed Fe^2+^ becomes the predominant aqueous Fe^II^ species, in line with the thermodynamic stability of different aqueous Fe^II^ species. Similarly, Fe^III^ initially exists as Fe­(OH)_3_ (aq), Fe­(OH)_4_^–^ and Fe­(OH)_2_^+^ (Figure 3d, h). Upon a decrease in the pH, the concentrations of Fe­(OH)_4_^–^ and Fe­(OH)_3_ (aq) reduce, whereas the concentration of Fe­(OH)_2_^+^ increases (Figure 3d, h). Our framework allows to track spatial and temporal changes in the speciation of Fe^II^ and Fe^III^ and the activity of each of the aqueous species as the pH and aqueous chemistry evolve.

**Figure 3:**
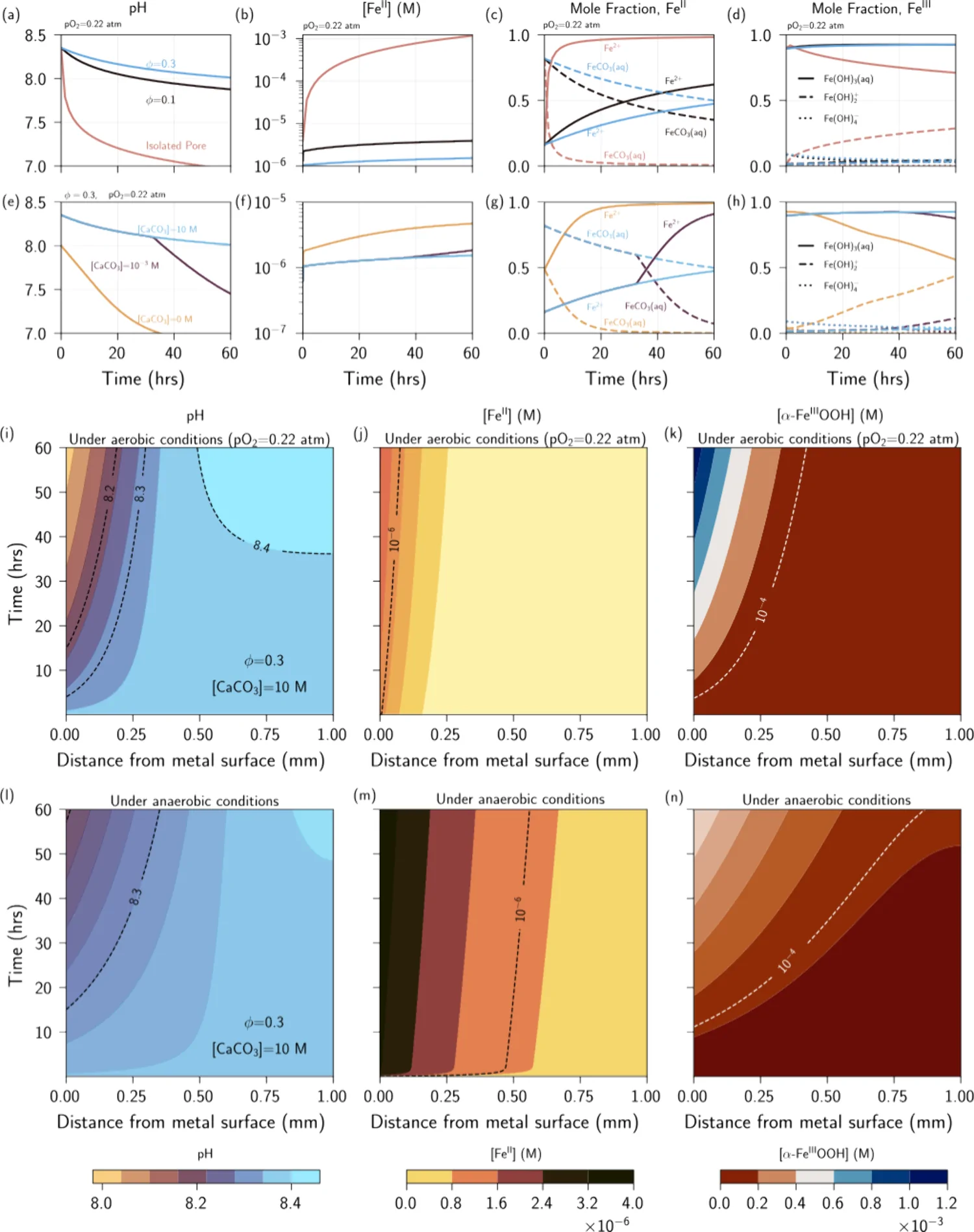
**Simulations of electrochemically dissolved iron in reactive porous media, revealing the temporal and spatial changes in pH, [Fe] as well as Fe speciation and precipitation as a function of the porosity, [CaCO_3_] and aeration condition:** (a–d) Modeled evolution of interfacial pH, [Fe^II^], speciation of Fe^II^, and speciation of Fe^III^, respectively, over time for three different average porosities of the porous medium (0.1, 0.3 and “isolated pore”; see Section 6.4) under aerobic conditions. (e–h) Modeled interfacial pH, [Fe^II^], speciation of Fe^II^, and speciation of Fe^III^ as a function of time and the amount of CaCO_3_ (10 M, 0.001 M and 0 M) surrounding the pore network under aerobic conditions. Here, the average porosity is constant and equivalent to 0.3. (i–n) Spatial and temporal evolution of pH, [Fe^II^] and the solid iron (hydr)­oxide (α-FeOOH, goethite) precipitated in aerobic and anaerobic conditions, respectively. For (i)–(n), both the average porosity and the amount of CaCO_3_ surrounding the pore network are fixed to 0.3 and 10 M, respectively. As the [Fe^III^] is governed by the solubility of goethite, the [Fe^III^] is in the order of 10^–13^ mol/L (see Figure S8 and Figure S9).

While the interfacial chemistry is important, the processes occurring further away from the metal surface are equally relevant for assessing the long-term corrosion behavior of the system.^44,49^ Owing to the slower rate of Fe^II^ oxidation to Fe^III^ under anaerobic environments,^2^ model results demonstrate higher [Fe^II^] at any point in space and time along the distance of 1 mm away from the metal surface under anaerobic conditions (Figure 3j) than aerobic conditions (Figure 3m). As a result (and in agreement with experimental observations^41^), the precipitation of Fe^III^ (hydr)­oxide (from aqueous Fe^III^)—goethite—is predicted to occur further away from the metal-porous medium interface in anaerobic environments, albeit at lower quantities when compared to the case of aerobic conditions (Figure 3k, n). Similar observations have been made experimentally for steel embedded in bentonite host rocks,^45,50,51^ providing qualitative validation of our reactive-transport framework. Aerobic conditions at the steel-bentonite interface promote precipitation of iron (hydr)­oxides in the interfacial region and anaerobic conditions lead to diffusion of Fe^II^ and the precipitation of iron (hydr)­oxides further away from the metal surface.^45,50,51^ Consequently, the reduction in the pH of the aqueous phase over time (lower than the initial pH of 8.4) within the porous medium is restricted to ∼300 μm within the first 60 h in the case of aerobic conditions (Figure 3i). Conversely, the pH of the aqueous phase in anaerobic conditions is lowered all along the distance of 1 mm away from the metal-porous medium interface (Figure 3l). As we assume Fe^III^ solid phases to precipitate instantaneously, the concentration of Fe^III^ (Figure S9) is dictated by the pH dependent solubility of the precipitating solid phase—goethite.

Given the empirical nature of existing models, such as those used for predicting the corrosion of steel in soil or cementitious media,^39,40,45^ we view the proposed model framework as a potential foundation for the development of more fundamental and mechanistic models.

### 3.2 Case II: Speciation of Manganese in Natural Waters

Manganese (Mn) plays an important role in natural coastal aqueous ecosystems, where it serves as a mediator in several redox cycles of elements such as iron, oxygen, sulfur, carbon, nitrogen, among others.^52,53^ Though Mn^IV^ (in the form of a solid oxide phase) is the thermodynamically most favored state in near-neutral aqueous environments, Mn^II^ and in particular, Mn^III^ constitute a large fraction of the soluble Mn-pool.^54,55^ The oxidation of Mn^II^ to Mn^IV^ and the reduction of Mn^IV^ to Mn^II^ involves the formation of a Mn^III^ intermediate that could either disproportionate or readily complexes with ligands (depending on the pH).^9,10,12,54,56,57^ Mn^III^ is an important redox species that can act as an oxidant or reductant and is responsible for the existence of suboxic zones in estuaries, oceans and sedimentary porewaters of lakes.^10^ The reactions surrounding the redox cycling of Mn in marine waters comprise several oxidative and reductive pathways (photochemical, biological, inorganic, pO_2_, surface-catalyzed, pH, among others) each affecting the reactivity of Mn and other biogeochemical cycles.^23,58–60^ In this context, we apply our reactive transport framework to study the speciation of Mn along a 30-m-deep water column (Figure 2b), supersaturated in CaCO_3_. We investigate scenarios of elevated levels of pCO_2_ in the atmosphere, potentially originating from multiple sources such as increased fossil fuel combustion, leakage of captured CO_2_, or microbial emissions.

We consider an initial distribution of Mn^II^ along the depth, similar to those profiled in the literature^61,62^ (Figure S2b). At the surface of the water column, we suppose an inward flux of Mn^II^ - equivalent to 10^–11^ mol/s/m^2^ that mimics natural processes such as lithogenic dust deposition.^12,61^ Here, we assume the activity of dissolved oxygen to vary spatially and decreases with depth (as reported in Grengs et al.,^63^ assumed constant throughout the simulation period) (Figure S2c). We consider three concentrations of CO_2_, each corresponding to a partial pressure of 10^–3.5^ atm, 10^–2.5^ atm and 10^–1.5^ atm. The entirety of the water column is assumed to be in equilibrium with pCO_2_. To observe the long-term implication of elevated CO_2_ levels, we simulate a time period of 340 days. We use commonly reported oxidation and reduction kinetic constants^11,12^ as a function of pH and pO_2_ (Figure S2a) to consider oxidation of Mn^II^ and reduction of Mn^III^, respectively, along with other transient processes such as diffusion and advection. This enables us to simultaneously solve for speciation of both Mn^II^ and Mn^III^, as a function of depth and time. Given the lack of thermodynamic data and redox rate laws concerning aqueous Mn^IV^ species, we currently cannot predict its speciation, activity or the amount of solid Mn^IV^ phases formed. Thus, Mn^IV^ is excluded in this example.

The results show that as Mn^II^ enters the water column, the higher pO_2_ at shallow depths facilitates the faster oxidation of Mn^II^ to Mn^III^ (Figure 4a, b, c). Whereas at lower depths, the slower oxidation of Mn^II^ and rapid reduction of Mn^III^, limits the activity of Mn^III^ and only causes negligible changes in the initial distribution of Mn^II^. Over time, the diffusion of Mn^II^ and Mn^III^ increases their respective activities along the depth of the water column. Similar to the Fe^II^–Fe^III^ system discussed in Section 3.1, our reactive transport framework allows us to map the influence of hydrolysis, speciation and precipitation of Mn^II^ and Mn^III^ on the pH across the entire spatial domain. Over the course of 340 days, the concentration of both Mn^II^ and Mn^III^ always remains below the solubility of solid Mn^II^ and Mn^III^ phases. Therefore, our model does not predict the precipitation of solid phases. Additionally, the high acid-neutralizing capacity of CaCO_3_ (s) considered here does not allow for the pH to change across the time and spatial domains we investigated.

**Figure 4:**
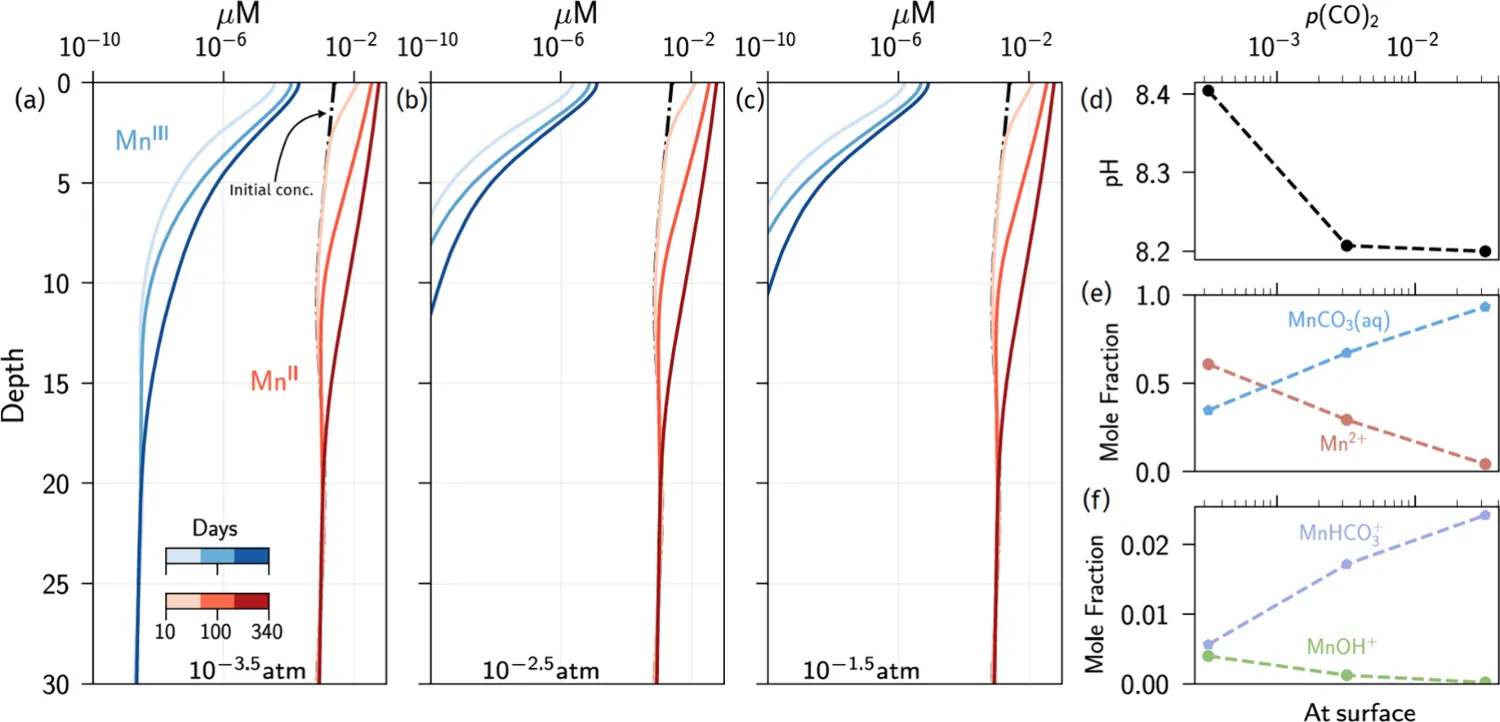
**Influence of elevated levels of CO_2_ concentration on Mn speciation in natural waters:** Distribution of Mn^II^ (red) and Mn^III^ (blue) along the depth of the water column for three different pCO_2_, (a) 10^–3.5^ atm, (b) 10^–2.5^ atm and (c) 10^–1.5^ atm at 10 days, 100 days and 340 days. Effect of pCO_2_ on (d) pH and (e, f) the mole fractions of relevant Mn^II^ complexes, after a period of 340 days and for the spatial location at the surface of the water column. The black dashed line represents the initial (*t* = 0) concentration of Mn^II^ as a function of depth (Figure S2b). A 0.1 M concentration of CaCO_3_ is assumed for all cases.

From our simulations we infer that an increase in the pCO_2_ in the atmosphere results in a decrease in the pH of the aqueous system (Figure 4d). While the reduction in the pH does not seem to influence the [Mn^II^] along the depth for the three pCO_2_ studied here (Figure 4a, b, c), speciation of Mn^II^ changes significantly. Mn^2+^ and MnCO_3_ (aq) constitute for roughly 60% and 40% of the total Mn^II^, respectively, when the pH is ∼8.4 (when pCO_2_ = 10^–3.5^ atm) (Figure 4e). At higher pCO_2_, the overall solubility of aqueous Mn^II^ is controlled by the solubility of MnCO_3_ (aq), and Mn^2+^ exists in minor quantities (Figure 4e). MnHCO_3_^+^ and MnOH^+^ exist in trace quantities (Figure 4f), and do not contribute significantly to the overall solubility of Mn^II^ in the pH range of 8.2–8.4. Finally, our model indicates (Figure 4a–c) that elevated pCO_2_ can decrease the amount of mobile Mn^III^ in natural waters with similar composition as assumed here. At a lower pH, the decreased rate of Mn^II^ oxidation in conjunction with a higher rate of Mn^III^ stabilizes Mn^II^ species in solution.

## 4 Discussion

Reactive transport modeling has matured into a diverse set of methodological approaches that enable coupling of transport processes with equilibrium thermodynamics and kinetics across a wide range of geochemical systems.^32^ Prominent frameworks—including PHREEQC-based,^22^ TOUGHREACT,^29^ CrunchFlow,^28^ MIN3P,^31^ PFLOTRAN,^27^ and related codes—share a common conceptual foundation rooted in the law of mass action for determining speciation and phase stability. These models have proven highly effective in near-equilibrium systems and in applications where redox transformations can reasonably be assumed to track thermodynamic constraints at the grid-cell scale.

Within this established modeling landscape, redox processes are commonly represented either as equilibrium reactions or as kinetic reactions formulated with an affinity term relative to equilibrium. While this formulation ensures internal thermodynamic consistency and often facilitates numerical stability, it implicitly assumes that redox reactions occurs on time scales that are short relative to transport. Therefore, redox distributions are typically constrained primarily by equilibrium thermodynamics, and intermediate oxidation states are transient unless explicitly protected through selective suppression, reaction partitioning, or pathway constraints defined by the user.

Gibbs free energy minimization (GEM)-based reactive transport approaches^18,64,65^ address some of the conceptual and numerical challenges associated with reaction-based implementations, particularly in complex multicomponent systems. By determining equilibrium states through global energy optimization, GEM avoids ambiguities related to reaction selection, redox potential formulation, and bookkeeping of mass-action constraints. However, GEM remains an equilibrium construct by design and therefore tends to predict instantaneous reorganization of redox species toward thermodynamically stable configurations. As such, it is best suited for defining equilibrium bounds and phase stability, rather than for describing kinetically limited redox evolution.

The framework developed in this work is intended to operate alongside, rather than in place of, these established methodologies. Its distinguishing feature is the explicit separation of redox reactions from equilibrium thermodynamic calculations. Redox transformations are treated as transient kinetic processes that evolve in space and time through competition with transport, using experimentally constrained bulk rate laws. At the same time, Gibbs free energy minimization is retained to resolve aqueous speciation and phase stability within each oxidation-state subsystem under a shared background chemistry.

This separation allows the framework to address regimes in which redox transformations are kinetically controlled, such as corrosion environments, suboxic waters, and systems involving mixed-valence transition metals, without abandoning the thermodynamic rigor that underpins established reactive transport models. Importantly, the approach does not seek to capture all scales or processes simultaneously; rather, it targets a specific modeling gap between fully equilibrium descriptions and highly detailed, species-resolved kinetic formulations that often suffer from parameter uncertainty.

These advantages come with trade-offs. The approach does not enforce global free energy minima across oxidation states and requires iterative coupling between parallel equilibrium solvers, increasing computational cost with the number of redox states considered. Consequently, the framework is not intended to replace equilibrium-based or fully kinetic models, but to complement them in regimes where redox kinetics and transport phenomena dominate system behavior. The framework expands the range of redox-sensitive systems that can be addressed in a mechanistically consistent manner, thereby, contributing to the broader reactive transport modeling ecosystem.

## 5 Outlook

We set out the fundamental basis for a methodology to gain insights into the evolution of the aqueous chemistry of anthropogenic and natural environments. This opens up several avenues for future research. The innate flexibility within our framework allows future work to integrate key transient processes such as the sorption of aqueous species onto solid surfaces, transformations of solid phases from thermodynamically less stable to more stable forms, and the influence of precipitation/dissolution on the pore structure, without compromising on the thermodynamic equilibrium calculations of the aqueous and solid phases of metals in several oxidation states. Moreover, we consider it a major advantage that our framework can also account for the precipitation of solid phases containing metals in mixed oxidation states, for example, Fe^II^ and Fe^III^ in Fe_3_O_4_ (s) or Mn^II^, Mn^III^ and Mn^IV^ in MnO_*x*_ (s)/Mn_3_O_4_ (s). We propose to take into account the precipitation of such solid phases along with other transient processes, provided availability of accurate experimentally determined rate laws. As with most reactive transport models, predicting long-term behavior is computationally intensive, particularly when large time steps are required. This presents a challenge that could be addressed by developing more efficient and innovative approaches to speed up thermodynamic calculations.^66,67^

While our reactive framework can provide insights on the evolution of redox sensitive aqueous chemistry, a major challenge lies in experimentally validating the simulation results. For example, within a porous media such as soil or concrete, it is nearly impossible to measure aqueous Fe^II^ or Fe^III^ below a certain concentration; or even discern the spatial and temporal fraction of Fe^II^ or Fe^III^ in the aqueous/solid medium. It is equally critical to ensure the preservation of redox-sensitive species such as Mn^II^, Mn^III^, Fe^II^, Fe^III^ or Cr^III^, Cr^IV^, Cr^V^, Cr^VI^ during experimentation, and obtaining systematic experimental data is practically difficult and mostly unavailable in the literature. Ongoing efforts include conducting carefully designed experiments^68^ to allow for qualitative and quantitative validation of our reactive-transport framework.

Our reactive transport framework showcases a novel approach in addressing the challenges in mapping the spatial and temporal distribution of elements across multiple redox states in aqueous systems. This capability is particularly valuable in fields such as toxicity assessment, groundwater remediation, and corrosion, among others, where metals can exist in multiple oxidation states and significantly influence aqueous chemistry and transport behavior.

## 6 Methods

### 6.1 Operator-Split Algorithm for Thermodynamic Modeling

As experimental data are scarce on individual formation rates κ_*i*_ of different M^b^ complexes from M^a^ complexes, it is not possible to model the chemical reactions as a whole, that is, under partial equilibrium conditions.^26^ To overcome this problem, we propose using an operator-split approach to simulate chemical reactions in two separate but parallel chemical systems (see Figure 1). In the first system, we consider the speciation of M^a^ and its precipitation, and in the second system, we consider the speciation and precipitation of M^b^. We consider the oxidation of M^a^ to M^b^ by introducing an auxiliary variable [M^b^], which represents the sum of all species in the oxidation state *b*. Similarly, to account for the reduction of M^b^ to M^a^, we consider an auxiliary variable [M^a^], which represents the sum of all species in the oxidation state *a*. The formation of each of the auxiliary variables is kinetically controlled with an overall rate constant κ that is representative of the combined formation rates of each M^b^ complex i.e. κ = *f*(κ_1_, κ_2_, κ_3_...κ_*n*_). Our choice is motivated by the fact that in experiments, the parameter κ is measurable instead of the individual parameters κ_*i*_^2^. We use an iterative approach to solve the two systems. Initially, the focus is on the first chemical system, which involves the equilibrium speciation and precipitation of M^a^. We employ the Gibbs free energy minimization principle for this purpose.^21^ The pH value obtained from this process, along with the equilibrium amount of background species (excluding M species), serves as the initial condition for the second chemical system. The second system is then solved to determine the speciation and precipitation of M^b^. At this point, the pH value obtained from the second system is checked. If the pH values of the first and second systems are within a specified tolerance (ΔpH < 10^–6^),^17^ it is assumed that the two systems have reached equilibrium. However, if the pH of the second system lies outside the specified tolerance criteria, the background species concentrations from the equilibrated second system are used as input for the first system, which is then re-equilibrated in the next iteration. This process is repeated until the pH values of both systems are within the chosen tolerance. Figure S3 shows a schematic representation of the operator-split algorithm for the thermodynamic modeling. It is worth noting that the proposed operator-split approach is robust, and the order in which the systems are solved does not impact the results.

### 6.2 Using Gibbs Minimization for Solving a Chemical System

For a given chemical system, the Gibbs minimization problem is formulated as

$$\underset{n}{\min}G=n_{i}\mu_{i}\mathrm{subject to}\{\begin{array}{l} A_{ij}n_{j}=b_{i}\\ n_{j}\geq 0\\ {\mathrm{CO}}_{2}=p\mathrm{atm} \end{array}$$ 1

where *n*_*i*_ is the moles of species, μ_*i*_ is the chemical potential of *i* ^*th*^ species, *A*_*ij*_ is the stoichiometric coefficient of *i* ^*th*^ species in *j* ^*th*^ reaction and *b*_*j*_ is the moles of *j* ^*th*^ element. The chemical potential function of the *i* ^*th*^ species is given as μ_*i*_(*n*) = μ_*i*_^*o*^ + RT ln­(*a*_*i*_(*n*)), where μ_*i*_^*o*^ is the standard chemical potential function and *a*_*i*_ is the activity function of the *i* ^*th*^ species. The activity of the *i* ^*th*^ species is defined as ln­(*a*_*i*_) = ln­(ψ_*i*_) + ln­(*x*_*i*_) + ln­(*c*), where for aqueous species ψ_*i*_ is equal to the activity coefficient γ_*i*_ and for gaseous specie ψ_*i*_ represents the fugacity coefficient. Similarly, *c* is a constant with a value of 1 for aqueous species and a value of c = P/P° for gaseous species. We employ the ideal activity model to calculate the coefficients of aqueous activity and gaseous activity.^21,26^ The oxidation and reduction kinetic rate constants are dependent on the solution’s pH and the oxygen state (aerobic or anaerobic) of the system. We use Reaktoro^21^ to solve the constrained Gibbs free energy minimization problem.

### 6.3 Coupling Diffusion Process with Chemical Reactions

We couple the proposed operator-split approach with a multispecies diffusion process to simulate and understand the complex mechanisms at the metal surface. The diffusion process is simulated using finite element based FEniCS computing platform. We employ a sequential noniterative approach^18,19^ to couple the chemical and diffusion processes. The coupled diffusion equations for M^a^ aqueous complexes, M^b^ aqueous complexes and auxiliary variables, [M^a^] and [M^b^], read

$$\underset{i}{\sum }{Ṁ}_{i}^{a}\left(x,t\right)=\underset{i}{\sum }\nabla D_{M_{i}^{a}}\cdot \nabla M_{i}^{a}\left(x,t\right)-\kappa_{\mathrm{ox}}\underset{i}{\sum }M_{i}^{a}\left(x,t\right)$$ 2

$$\underset{j}{\sum }{Ṁ}_{j}^{b}\left(x,t\right)=\underset{j}{\sum }\nabla D_{M_{j}^{b}}\cdot \nabla M_{j}^{b}\left(x,t\right)-\kappa_{\mathrm{red}}\underset{j}{\sum }M_{j}^{b}\left(x,t\right)$$ 3

$${Ṁ}^{b}\left(x,t\right)=\nabla D_{M^{b}}\cdot \nabla M^{b}\left(x,t\right)+\kappa_{\mathrm{ox}}\overset{m}{\underset{i=1}{\sum }}M_{i}^{a}-\kappa_{\mathrm{red}}M^{b}$$ 4

$${Ṁ}^{a}\left(x,t\right)=\nabla D_{M^{a}}\cdot \nabla M^{a}\left(x,t\right)-\kappa_{\mathrm{ox}}M^{a}+\kappa_{\mathrm{red}}\overset{n}{\underset{j=1}{\sum }}M_{j}^{b}$$ 5

where *D* represents the diffusion coefficient for each of the species in the system. For the other aqueous species (i.e., background species in our framework), we employ the above equation for the diffusion process but without any source or sink term. We perform a sensitivity analysis to ensure that the time-step size Δ*t* does not affect the reactive transport modeling; see Figure S4. Based on the sensitivity analysis, we chose a time step of 5 s for the simulations in the paper. The concentration of each species (both aqueous and solid) is expressed as moles per liter. We compute the total number of moles of each species in a finite element and divide it by the total volume of that spatial finite element.

### 6.4 Iron in Porous Media

We perform reactive transport simulations over a length of 1 mm along the steel-porous medium interface. We model all reactive-transport processes in a one-dimensional space, with a finite element discretization of 2.5 μm. We assume that the iron corrodes at a constant rate of 1 μA/cm^2^ leading to a constant flux of Fe^2+^ ions from the steel surface. The rate at which Fe^II^ oxidizes to Fe^III^ depends on the pH, pO_2_ according to the values reported in the literature.^2^ We approximate the complex pore structure of a porous medium and assume three microstructural scenarios at the steel-porous medium interface (Figure S1a), with different average porosities. In the first two scenarios, we consider two average porosities, namely 0.1 and 0.3, perpendicular to the steel (Figure S1a). The third scenario considers the case of a significantly small pore at the steel-porous medium interface completely filled with an aqueous solution. In other words, this represents a thin pore along the length of the steel, where the diffusion of any aqueous species is not considered (we assume *D* to be 0 in eqs 3–5), and we term this an “isolated pore” (or could be considered a “crevice” in the field of corrosion science) (Figure S1a). The solids surrounding the pores are solely constituted of a reactive species—CaCO_3_ (s)—in equilibrium with the aqueous phase and can congruently dissolve in case of changes in the pH of the electrolyte. We vary the initial amount of CaCO_3_ (s) present in our simulations from 10 mol/L to 0.001 mol/L to 0 mol/L to study the influence of the acid-neutralizing capacity of CaCO_3_ (s) surrounding the pore network. While assessing the influence of the acid-neutralizing capacity of CaCO_3_ (s), we fix the porosity to a value of 0.3. As the concentration of dissolved oxygen can influence the rate at which Fe^II^ oxidizes, we consider the porous medium to be either aerobic or anaerobic. The influence of oxygen on the spatial and temporal evolution of the chemistry of the porous medium is assessed using experimentally determined pseudo-first order kinetic constants of Fe^II^ oxidation in aerobic and anaerobic environments^2^ (Figure S1c). While assessing the influence of oxygen, we fix the amount of CaCO_3_ surrounding the pores to 10 M, and the average porosity to 0.3.

### 6.5 Manganese in Natural Waters

We consider a water column 30 m in depth. We model all reactive-transport processes in a one-dimensional space, with a finite element discretization of 0.1 m. We assume that the partial pressure of O_2_ decreases exponentially as a function of depth from the water surface given as pO_2_(*y*) = 0.22 e^–*y*/5^_,_ where 0.22 is the atmospheric pressure of O_2_ and *y* is the depth from the water surface (Figure S2c). We assume that the partial pressure of CO_2_ does not change along the depth of the water column. Additionally, we assume the water column is always supersaturated with respect to CaCO_3_ (s). In Section 3.2, we use 3 different values for pCO_2_ and assume that the entire water column is in equilibrium with elevated pCO_2_ and thus the entire column has the same pCO_2_. The overall pseudo first-order oxidation and reduction kinetic rate constants are determined from the experimentally recorded values from the literature^11,12^ and is a function of the pH and pO_2_ (Figure S2a).

## Supplementary Material



## Data Availability

The code developed and data sets generated during and/or analyzed during the current study are available in the Gitlab.ethz.ch repository, https://gitlab.ethz.ch/smec/papers-supp-info/2025/redox-reactive-transport.

## Notes

Additional details on methodology and reactive transport modeling; tabulated thermodynamic data; supporting figures; supplementary notes on limitations of the framework (PDF)
