# Supplementary material for: Beyond Equilibrium: Tracing Redox-Driven Pathways That Shape Aqueous and Mineral Chemistry
Source: Environ Sci Technol. 2026 Oct 14;60(42):30210–20. doi: 10.1021/acs.est.6c08480 (PMC13718617; doi:10.1021/acs.est.6c08480)
Supplement: Supplementary file 1 [file es6c08480_si_001.pdf]

Supplementary information:

Beyond Equilibrium: Tracing redox-driven pathways that shape aqueous and mineral chemistry

Shishir Mundra[1*], Mohit Pundir[1], Barbara Lothenbach[2], David S. Kammer[1] and Ueli M. Angst[1]

[1]Institute for Building Materials (IfB), ETH Zurich, Laura-Hezner-Weg 7, 8093 Zurich, Switzerland

[2]EMPA, Laboratory for Concrete and Asphalt, CH – 8600, Dübendorf, Switzerland

*Corresponding author: smundra@ethz.ch

Table of Contents

1 Supplementary Note 1: Methods 2

2 Supplementary Note 2: Thermodynamic databases 3

3 Supplementary Note 3: Schematic representation of operator-split thermodynamic modelling 5

4 Supplementary Note 4: Validation of time-step for SNIA approach 5

5 Supplementary Note 5: Interfacial chemistry in the context of steel corrosion in porous media such as concrete 6

5.1 Acid neutralising capacity as a function of [$CaCO_3$] 7

5.2 Influence of [$CaCO_3$] and oxygen on pH buffering 8

5.3 Solubility of Fe^{II} in the presence of $CaCO_3$ 10

5.4 Fe^{III} at the metal-porous medium interface and further away from the metal surface 11

5.5 Framework limitations 12

1 Supplementary Note 1: Methods

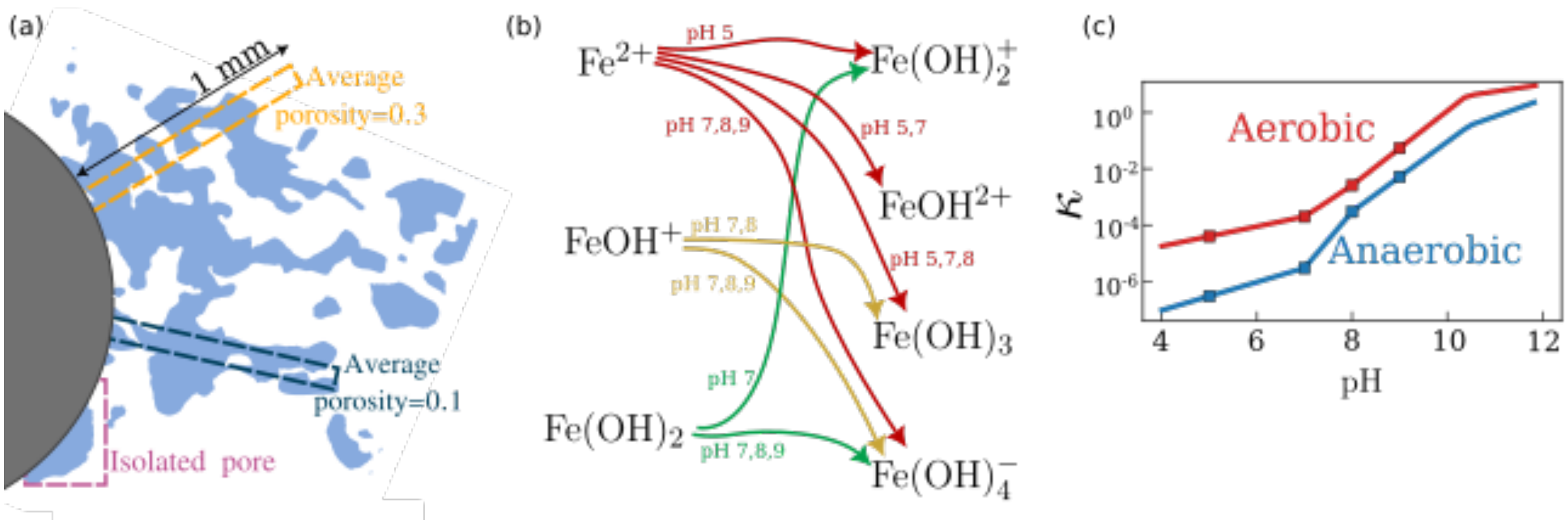

Figure S 1: (a) Schematic representation of the steel concrete interface, with three average porosities. (b) Oxidation schematic with different Fe^{II} and Fe^{III} species[1]. (c) Pseudo-first order oxidation kinetics (in sec^{-1} of Fe^{II} as a function of pH (aerobic and anaerobic)[1].

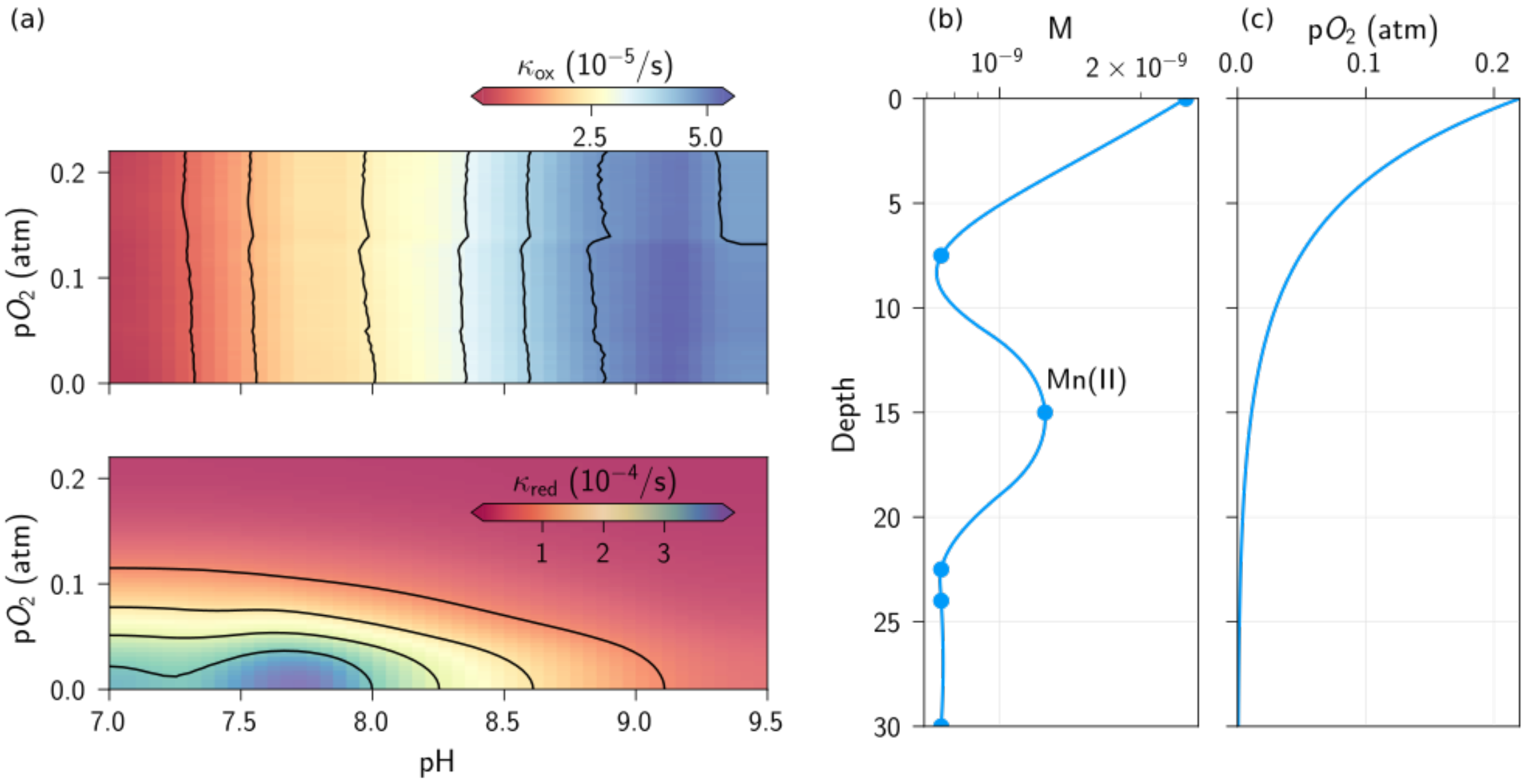

Figure S 2: (a) Mn^{II} oxidation and Mn^{III} reduction kinetics interpolation vs. pH and pO_2 (data from the literature[2,3]) (b) Initial conditions of Mn^{II} distribution in oceans[4] (c) Oxygen concentration vs. depth[4]

2 Supplementary Note 2: Thermodynamic databases

Table S 1: Thermodynamic data for the solid, aqueous and gaseous species considered in this study for the Fe-C-H_2O system[5–12].

Species	MW (g/mol)	$\Delta_f G^\circ_m$, kJ mol^{-1}	$\Delta_f H^\circ_m$, kJ mol^{-1}	S°_m, J mol^{-1} K^{-1}	$C^\circ_{p,m}$, J mol^{-1} K^{-1}	V°, J bar^{-1}	Ref.
α - Fe_0	55.8450	0	0	27.085 ± 0.160	25.084 ± 0.500	0.7092 ± 0.0004	5, 6
Fe^{2+}	55.8450	-90.719 ± 0.641	-90.295 ± 0.522	-102.171 ± 2.770	-23.000 ± 10.00	-2.264	5
Fe^{3+}	55.8450	-16.226 ± 0.650	-50.056 ± 0.973	-282.404 ± 3.927	-108.000 ± 20.00	-3.779	5
**Fe(II) hydrolysis products**							
$Fe(OH)^+$	72.8523	-273.996 ± 0.859^a	-321.525 ± 0.104	-29.628 ± 4.851	63.061	-1.671	7
$Fe(OH)_2$ (aq)	89.8597	-447.798 ± 0.791^a	-546.555 ± 1.131	31.930 ± 4.941			7
$Fe(O)$ (aq) (+ H_2O (l) = $Fe(OH)_2$ (aq))	71.8444	-210.694 ± 0.792	-260.725 ± 1.131	-38.020 ± 4.940	-0.013	-1.649	7, 8
$Fe(OH)_3^-$	106.867	-615.492 ± 1.077	-807.485 ± 2.264	-47.406 ± 8.579			7
FeO_2H^- (+ H_2O (l) = $Fe(OH)_3^-$)	88.8518	-378.388 ± 1.077	-521.655 ± 2.264	-117.356 ± 8.579	92.844	-1.347	7–9
**Fe(III) hydrolysis products**							
$FeOH^{2+}$	72.8523	-240.772 ± 0.661^a	-292.586 ± 1.144	-109.344 ± 5.503	-33.693	-2.534	7
$Fe(OH)_2^+$	89.8597	-457.841 ± 0.869^a	-550.200 ± 1.00	-11.848 ± 4.891			7
FeO^+ (+ H_2O (l) = $Fe(OH)_2^+$)	71.8444	-220.737 ± 0.870	-264.300 ± 1.00	-81.798 ± 4.890	-200.942	-4.202	7–9
$Fe(OH)_3$ (aq)	106.867	-657.557 ± 1.625^a	-761.246 ± 4.899	183.423 ± 17.61			7
FeO_2H (aq) (+H_2O (l) = $Fe(OH)_3$ (aq))	88.8518	-420.453 ± 1.625	-475.416 ± 4.899	113.473 ± 17.61	-312.138	-0.721	7, 8
$Fe(OH)_4^-$	123.874	-841.348 ± 1.474^a	-1046.58 ± 2.052	76.237 ± 9.067			7
FeO_2^- (+ 2H_2O (l) = $Fe(OH)_4^-$)	87.8438	-367.140 ± 1.474	-474.916 ± 2.052	6.287 ± 9.067	-234.928	0.045	7, 8
$Fe_2(OH)_2^{4+}$	145.705	-490.050 ± 1.362^a	-641.672 ± 9.698	-379.664 ± 33.48			7
$Fe_3(OH)_4^{5+}$	235.564	-961.133	-1234.49	-490.138			9
**Fe(II) chloride complexes**							
$FeCl^+$	91.298	-216.228 ± 4.613^a	-235.825 ± 1.848	7.563 ± 16.574	86.492	0.085	10
$FeCl_4^{2-}$	197.657	-584.735 ± 3.352^a	-687.215 ± 2.585	260.229 ± 7.571			10
**Fe(III) chloride complexes**							
$FeCl^{2+}$	91.298	-156.119 ± 0.873	-194.656 ± 4.703	-121.309 ± 16.04	14.828	-2.286	10
$FeCl_2^+$	126.751	-291.332 ± 1.442^a	-342.386 ± 8.864	13.596 ± 33.631	300.718	1.027	10
$FeCl_3$ (aq)	162.204	-415.870 ± 1.681	-494.666 ± 11.34	97.436 ± 50.866	368.217	3.594	10
$FeCl_4^-$	197.657	-536.870 ± 3.072	654.246 ± 11.44	144.922 ± 64.30			10
**Fe(II) carbonate complexes**							
$FeHCO_3^+$	116.862	-685.600	-776.3	36.550	231.409	0.818	10
$FeCO_3$ (aq)	115.854	-648.677 ± 1.285	-770.99	-62.000	-123.025	-1.723	10
$Fe(CO_3)_2^{2-}$	175.865	-1186.67 ± 1.293	-1384.72	128.000			10
**Solid Fe(II) hydroxides**							
$Fe(OH)_2$ (s) – White Rust	89.8597	-494.889 ± 5.069	-583.39	84.00	≈ 90.000	2.952	6, 10
**Solid Fe(III) hydroxide**							
$Fe(OH)_3$ (s) – 2-line ferrihydrite	106.867	-708.500 ± 2.000	-827.100 ± 2.000	127.600 ± 5.400	$\approx 152.000 \pm 5.00$	3.400	11
α-FeOOH (s) – Goethite	88.8518	-489.54 ± 2.000	-560.50 ± 2.000	59.700 ± 0.500	74.400 ± 0.40	2.090	10
**Solid Fe(II) carbonates**							
$FeCO_3$ (s) – Siderite	71.8444	-679.557 ± 0.917	-752.609 ± 0.895	95.537 ± 0.646	82.450 ± 2.000	2.938	10
**Calcium carbonate and related aqueous and solid species**							
$CaCO_3$ (s) – Calcite	100.086	-1129.176	-1207	92.7	81.9		9, 12
$Ca(OH)_2$ (s)	74.093	-897.013	-984.675	83.399	87.505	3.306	9, 12
Ca^{2+}	40.078	-552.79	-543.07	-56.48	-30.92	-1.844	9, 12
$CaOH^+$	57.085	-717.02	-751.65	28.03	6.05	0.576	9, 12
$CaCO_3$ (aq)	100.086	-1099.18	-1201.92	10.46	-123.86	-1.565	9, 12
$CaHCO_3^+$	101.094	-1146.04	-1231.94	66.94	233.70	1.333	9, 12
CO_3^{2-}	60.009	-527.900 ± 0.390	-675.230 ± 0.250	50.000 ± 1.000	-289.33	-0.06	9, 12
HCO_3^-	61.017	-586.845 ± 0.251	-689.930 ± 0.200	98.400 ± 0.500	-34.85	2.421	9, 12
CO_2 (aq)	44.009	-386.02	-413.84	117.57	243.08	3.281	9, 12
**Auxiliary species**							
Na^+	22.9897	-261.88	-240.28	58.41	38.12	-1.21	9, 12
Cl^-	35.453	-131.29	-167.11	56.74	-122.49	17.34	9, 12
O_2 (g)	31.9988	0	0	205.152 ± 0.005	29.378 ± 0.003	2478.970	9, 12
O_2 (aq)	31.9988	16.45	-12.24	108.95	234.13	30.50	9, 12
H_2 (g)	2.0159	0	0	13.680 ± 0.003	28.836 ± 0.002	2478.970	9, 12
H_2 (aq)	2.0159	17.73	-4.02	57.74	166.85	25.26	9, 12
CO_2 (g)	44.009	-394.39	-393.51	213.74	37.15		9, 12
H_2O (l)	18.0153	-237.140 ± 0.041	-285.830 ± 0.040	69.650 ± 0.030	75.351 ± 0.080	1.807	9, 12
H_2O (g)	18.0153	-228.582 ± 0.040	-241.826 ± 0.040	188.835 ± 0.010	33.609 ± 0.030	2478.970	9, 12
H^+	1.008	0	0	0	0	0	9, 12
OH^-	17.007	-157.220	-230.015 ± 0.040	-10.900 ± 0.200	-136.34	-4.71	9, 12

[a] Internally computed from $\Delta_r G^\circ_m = \sum_i \nu_i \Delta_f G^\circ_{m,i}$.

Table S 2: Thermodynamic data for the solid, aqueous and gaseous species considered in this study for the Mn-C-H_2O system [7,9,12,13].

Species	MW (g/mol)	$\Delta_f G^\circ_m$, kJ mol^{-1}	$\Delta_f H^\circ_m$, kJ mol^{-1}	S°_m, J mol^{-1} K^{-1}	$C^\circ_{p,m}$, J mol^{-1} K^{-1}	V°, J bar^{-1}	Ref.
Mn^{2+}	54.938	−230.538	−221.328	−67.781	−16.552	−1.753	9
Mn^{3+}	54.938	−84.935	−128.449	−309.62	−105.95	−4.1827	9
**Mn(II) hydrolysis products**							
$MnOH^+$	71.945	−407.273^a	−447.025	1.255	36.637	−1.207	7, 9
$Mn(OH)_2$ (aq)	88.95	-575.8 ± 1.2^a	-675.0 ± 2.6	35.1 ± 9.8			7, 9
$Mn(OH)_3^-$	105.93	-743.6 ± 2.6^a	-906.4 ± 3.1	55 ± 14			7, 9
$Mn(OH)_4^{2-}$	122.967	-901.2 ± 2.3^a	-1107.7 ± 5.2	142 ± 19			7, 9
**Mn(III) hydrolysis products**							
$MnOH^{2+}$	71.945	-324.4 ± 1.0^a	-262.90 ± 5.5	276 ± 19			7, 9
$Mn(OH)_2^+$	88.95	−565.842^a					9
**Mn(II) chloride complexes**							
$MnCl^+$	90.391	−365.310^a	−373.659	50.208	105.258	0.663	9
**Mn(III) chloride complexes**							
$MnCl^{2+}$	90.391	−218.783^a					9
**Mn(II) carbonate complexes**							
$MnCO_3$ (aq)	114.947	−786.489	−893.148	−12.259	−107.127	−1.366	13
$MnHCO_3^+$	115.954	−828.609	−918.253	44.810	266.159	1.437	13
**Solid Mn(II) phases**							
$Mn(OH)_2$ (s)	88.95	-615.7 ± 0.8	−695.4	99.2			9
MnO (s)	70.937	-362.9 ± 0.5	-385.22 ± 0.5	59.71 ± 0.4	44.762	1.322	9
$MnCO_3$ (s)	114.947	-818.13 ± 0.55	-891.91 ± 0.52	98.03 ± 0.1	81.533	3.1075	9, 13
**Solid Mn(III) phases**							
MnOOH (s)	87.945	-557.8 ± 2.0	−650.453		79.652		9
**Calcium carbonate and related species**							
$CaCO_3$ (s) – Calcite	100.086	−1129.176	−1207	92.7	81.9		9, 12
$Ca(OH)_2$ (s)	74.093	−897.013	−984.675	83.399	87.505	3.306	9, 12
Ca^{2+}	40.078	−552.79	−543.07	−56.48	−30.92	−1.844	9, 12
$CaOH^+$	57.085	−717.02	−751.65	28.03	6.05	0.576	9, 12
$CaCO_3$ (aq)	100.086	−1099.18	−1201.92	10.46	−123.86	−1.565	9, 12
$CaHCO_3^+$	101.094	−1146.04	−1231.94	66.94	233.70	1.333	9, 12
CO_3^{2-}	60.009	-527.900 ± 0.390	-675.230 ± 0.250	50.000 ± 1.000	−289.33	−0.06	9, 12
HCO_3^-	61.017	-586.845 ± 0.251	-689.930 ± 0.200	98.400 ± 0.500	−34.85	2.421	9, 12
CO_2 (aq)	44.009	−386.02	−413.84	117.57	243.08	3.281	9, 12
**Auxiliary species**							
Na^+	22.9897	−261.88	−240.28	58.41	38.12	−1.21	9, 12
Cl^-	35.453	−131.29	−167.11	56.74	−122.49	17.34	9, 12
O_2 (g)	31.9988	0	0	205.152 ± 0.005	29.378 ± 0.003	2478.970	9, 12
O_2 (aq)	31.9988	16.45	−12.24	108.95	234.13	30.50	9, 12
H_2 (g)	2.0159	0	0	13.680 ± 0.003	28.836 ± 0.002	2478.970	9, 12
H_2 (aq)	2.0159	17.73	−4.02	57.74	166.85	25.26	9, 12
CO_2 (g)	44.009	−394.39	−393.51	213.74	37.15		9, 12
H_2O (l)	18.0153	-237.140 ± 0.041	-285.830 ± 0.040	69.650 ± 0.030	75.351 ± 0.080	1.807	9, 12
H_2O (g)	18.0153	-228.582 ± 0.040	-241.826 ± 0.040	188.835 ± 0.010	33.609 ± 0.030	2478.970	9, 12
H^+	1.008	0	0	0	0	0	9, 12
OH^-	17.007	−157.220	-230.015 ± 0.040	-10.900 ± 0.200	−136.34	−4.71	9, 12

^{a} Internally computed from $\Delta_r G^\circ_m = \sum_i \nu_i \Delta_f G^\circ_{m,i}$.

3 Supplementary Note 3: Schematic representation of operator-split thermodynamic modelling

Figure S 3 shows the schematic representation of the operator-split algorithm for thermodynamic modelling. Although, the algorithm works in a *n*-dimensional space (where *n* is the total number of species in the chemical system), here for ease of understanding we have reduced it to a 2-dimensional space.

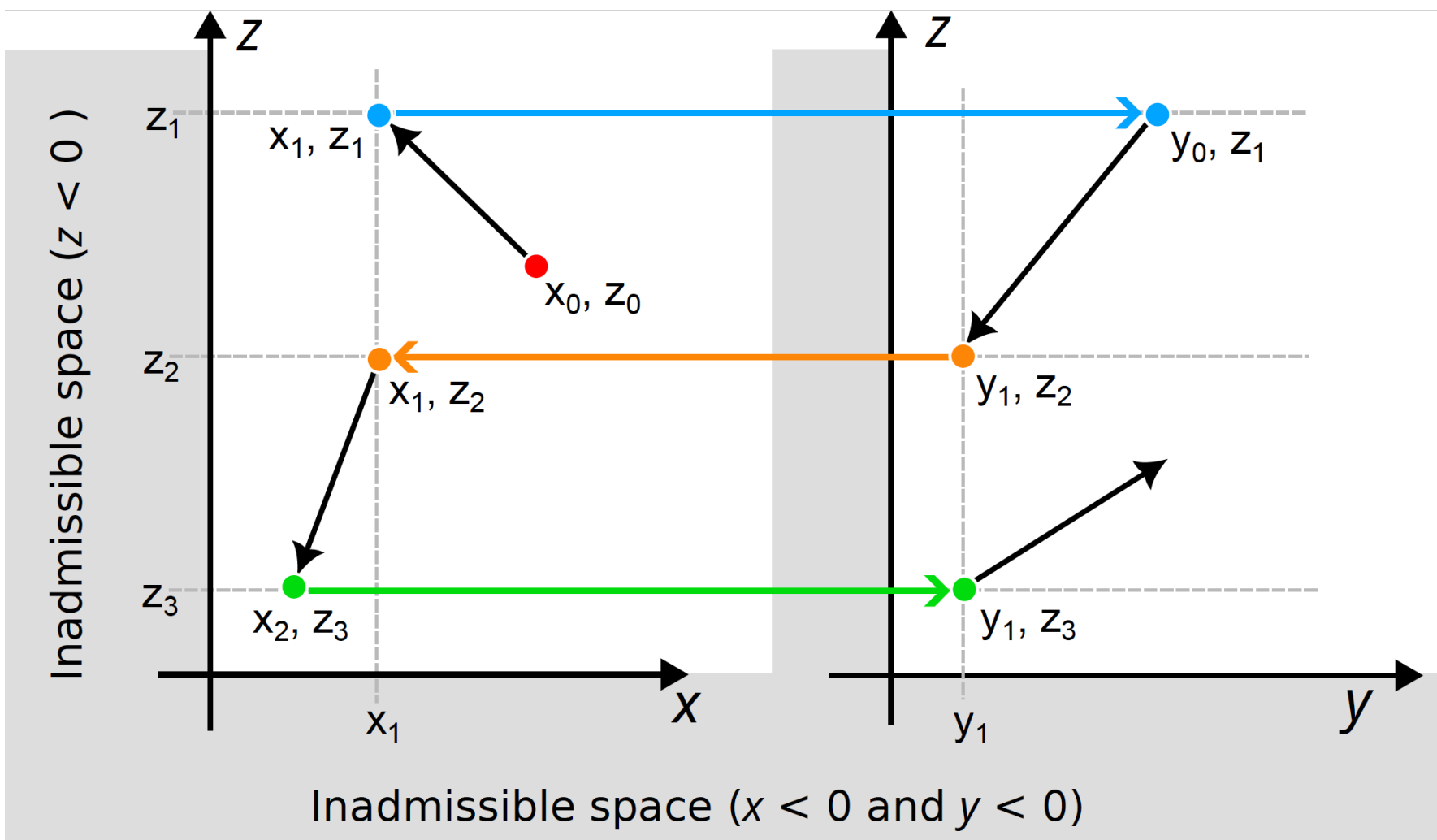

Figure S 3: Schematic representation of operator-split algorithm for thermodynamic modelling of multiple redox states. Here *x* refers to the total concentration of all M^a complexes, *y* refers to the total concentration of all M^b complexes and *z* refers to the total concentration of all the background species. For visual representation, we show the concentration of all background species as one axis, however, in reality the space is multi-dimensional with individual axes for each background species

4 Supplementary Note 4: Validation of time-step for SNIA approach

In our reactive transport framework, we employ a staggered non-iterative approach (SNIA) to couple the transient processes such as diffusion and kinetically controlled reactions with the non-transient processes such as chemical reactions at equilibrium. We performed a sensitivity analysis to find the optimal time step for temporal evolution. Figure S 4 shows the effect of time step on the spatial evolution of the concentration of the chemical species. We note that we hose the time step value for which the results are converged.

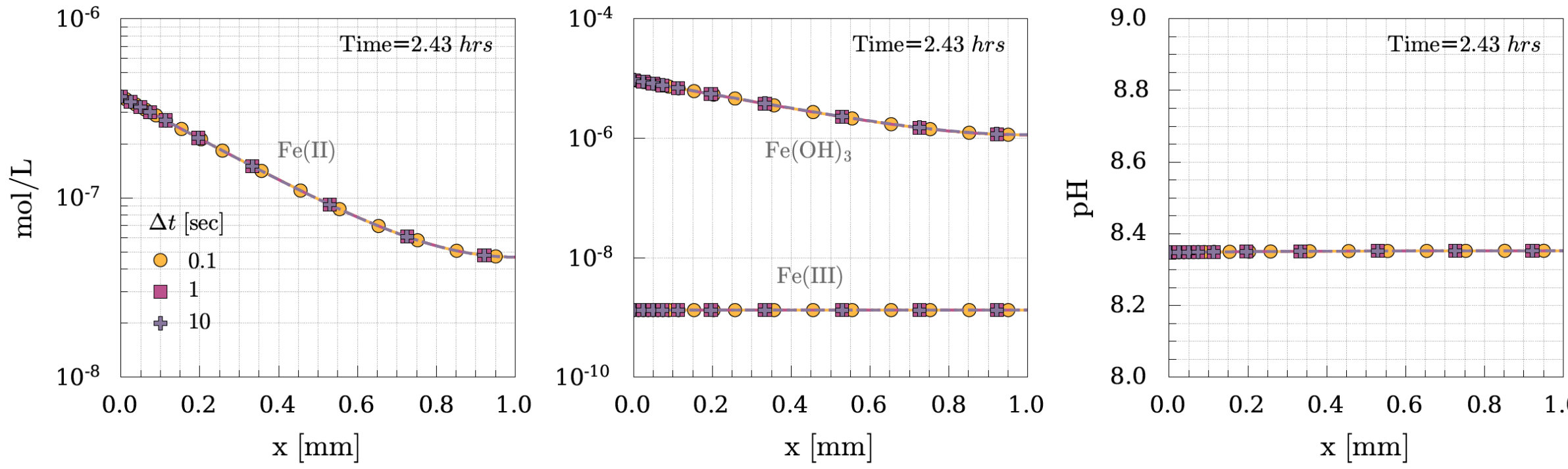

Figure S 4: Sensitivity analysis to investigate the effect of time step (0.1 sec to 10 sec) on the modelling of reactive transport using an SNIA approach

5 Supplementary Note 5: Interfacial chemistry in the context of steel corrosion in porous media such as concrete

Steel develops a robust thermodynamically stable passive film in highly alkaline (pH: > 12.5) solutions or in a porous medium such as concrete. The breakdown of steel passivity in such highly alkaline porous environments can be either local due to the action of aggressive species such as chlorides or uniform due to the lowering of the pH of the aqueous phase due to environmental conditions. When considering localised corrosion, the breakdown of passivity may cause a change in the local pH in the area of active corrosion. In concrete particularly, this change in the local pH might be buffered for a significantly long time due to the dissolution of $Ca(OH)_2$ (s) present in cementitious matrix. The carbonation of the concrete (i.e. decalcification of Ca-bearing cement hydrate phases and the precipitation of $CaCO_3$) and the associated loss of alkalinity (pH < 9) is often 'perceived' as a cause for the corrosion of the embedded steel reinforcement[14]. However, in the context of infrastructure corrosion, steel corrosion rates are generally only of concern when the internal relative humidity at the steel-concrete interface (SCI) is ≥ 80 – 95 % [15,16]. In the case of fully saturated and completely carbonated concretes, the corrosion of the embedded reinforcement may be sustained.

The sustained anodic dissolution of iron leads to the production of Fe^{2+} (Eq. S1) and the simultaneous cathodic generation of OH^- (the flux of $[OH^-]$ is twice that of $[Fe^{2+}]$). In the aqueous phase, Fe^{2+} may hydrolyse (Eq. S2) (depending on the pH) and may oxidise to Fe^{3+} or any of the Fe^{III} hydrolysis products (Eq. S3 and Eq. S4), releasing protons. Upon reaching the

solubility limit w.r.t. to different iron (hydr)oxides, both Fe^{II} and Fe^{III} can undergo precipitation as represented for the simple cases of $Fe(OH)_2$ and $Fe(OH)_3$ by Eq. S5 and Eq. S6, respectively, all causing changes in the chemistry of the aqueous phase. These changes in the aqueous chemistry influence the kinetics of oxidation, hydrolysis, speciation and precipitation of Fe. The ever-evolving chemistry may influence the rate at which the metal (iron in this case) corrodes.

$$Fe \rightarrow Fe^{2+} + 2e^{-} \quad \text{(Eq. S1)}$$

$$Fe^{2+} + mH_2O \rightarrow Fe(OH)_m^{2-m} + mH^{+} \quad \text{(Eq. S2)}$$

$$Fe(II) \rightarrow Fe(III) + e^{-} \quad \text{(Eq. S3)}$$

$$Fe^{3+} + nH_2O \rightarrow Fe(OH)_n^{3-n} + nH^{+} \quad \text{(Eq. S4)}$$

$$Fe^{2+} + 2H_2O \rightarrow Fe(OH)_2\ (s) + 2H^{+} \quad \text{(Eq. S5)}$$

$$Fe^{3+} + 3H_2O \rightarrow Fe(OH)_3\ (s) + 3H^{+} \quad \text{(Eq. S6)}$$

In fully carbonated concrete, the pore solution is in equilibrium with $CaCO_3$ (s) surrounding the pore network. Changes in the pH of the pore solution due to the reactions mentioned in Eq. S1 – Eq. S6, may be countered/buffered by the dissolution of $CaCO_3$ (s).

5.1 Acid neutralising capacity as a function of [$CaCO_3$]

The degree of super/under saturation with respect to $CaCO_3$ (s) influences the rate of change of pH, speciation of aqueous carbonate and the concentration of mobile metal cations. Figure S 5a and Figure S 5b show the influence of [$CaCO_3$] in anaerobic and aerobic environments, for the example of steel corrosion in concrete. The $CaCO_3$ (s) can dissolve to produce $HCO3^{-}$ and CO_3^{2-} to buffer any changes in the pH. Therefore, the acid neutralising capacity of the interfacial electrolyte is strongly dependent on the degree of super/under saturation w.r.t. $CaCO_3$; and a higher [$CaCO_3$] would yield a lower rate of acidification (Figure 3e – main manuscript). This is analogous to the influence of anthropogenic CO_2 on the dissolution of $CaCO_3$ in the oceans, where the degree of supersaturation of $CaCO_3$ as a function of the oceanic depth controls the acid neutralising capacity of the waters[17–23].

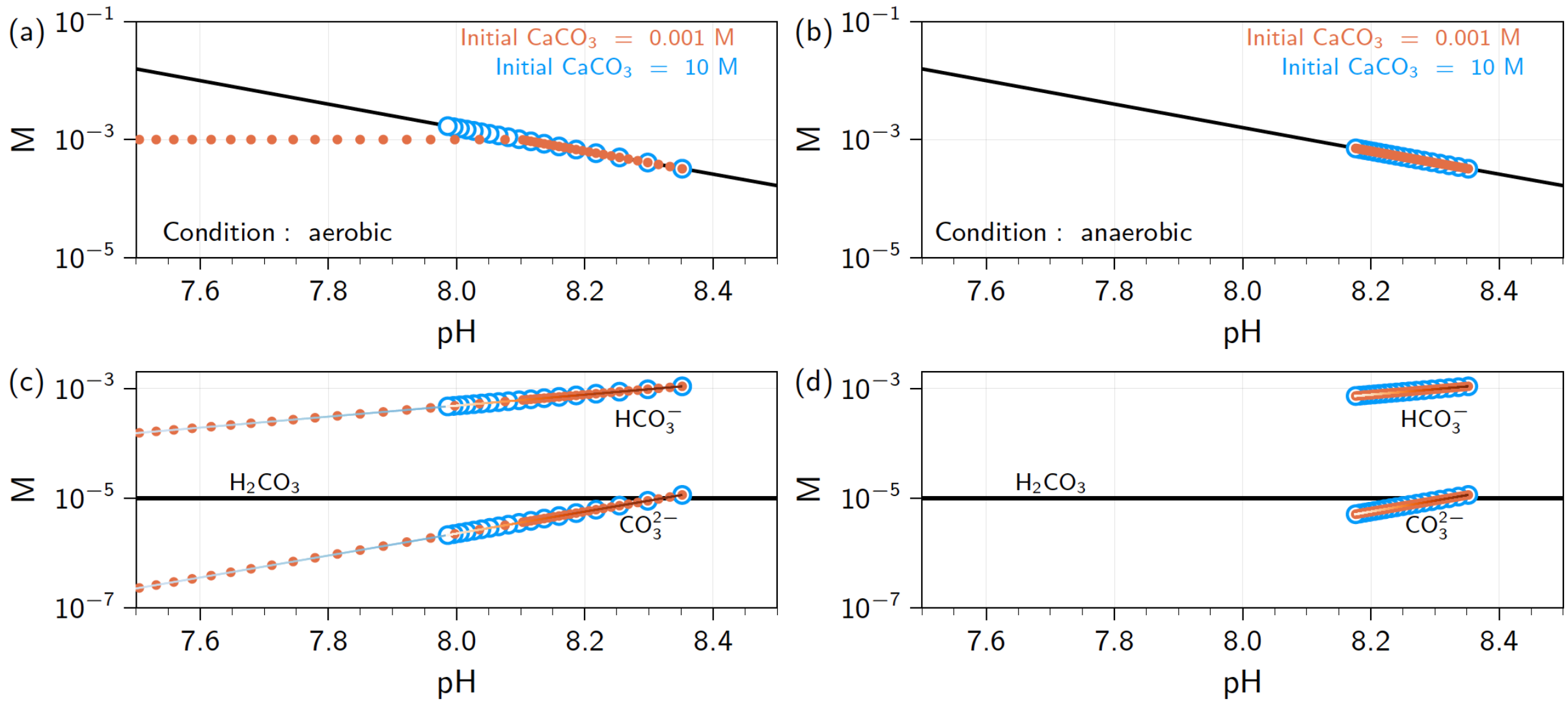

Figure S 5: The acid-neutralising capacity of $CaCO_3$ is shown through the solubility of $CaCO_3$ (s) and speciation of aqueous carbonate species under (a, c) aerobic and (b, d) anaerobic conditions. The blue and red markers indicate the pH and the corresponding speciation of aqueous carbonate species when the initial [$CaCO_3$] is 10 mol/L and 0.001 mol/L, respectively.

5.2 *Influence of [$CaCO_3$] and oxygen on pH buffering*

Let us look at the influence of [$CaCO_3$] surrounding the pore network on the pH buffering at the metal-porous medium interface. We assume that $CaCO_3$, formed as a result of the carbonation of the binder, can instantaneously dissolve into bicarbonate and carbonate anions, and their respective aqueous molar fractions depend on the prevailing pH. Therefore, we do not consider the kinetics of $CaCO_3$ dissolution as a function of the gradients in the pH, and speciation of aqueous inorganic carbon at the surface of the dissolving $CaCO_3$ (s). Regardless of whether the interfacial electrolyte is oxygenated or deoxygenated, the higher the amount of $CaCO_3$, the lower the rate at which the interfacial pH decreases (as seen in Figure S 6a, d). Over the course of 72 hours in aerobic and anaerobic electrolytes and when 10 mol/L $CaCO_3$ surrounds the interfacial electrolyte, Δ pH at the interface is ≈ ~ -0.4 and -0.25, respectively (Figure S 6a, d). Whereas for [$CaCO_3$] = 10^{-3} mol/L, Δ pH at the interface over 72 hours is ≈ -1.1 and -0.25, for aerobic and anaerobic conditions, respectively (Figure S 6a, d). In the absence of $CaCO_3$, the pH reduces by approximately 2 units in aerobic conditions and 0.8 units in anaerobic conditions, over the same duration (Figure S 6a, d). Upon a decrease in the interfacial pH (due to corrosion of steel and associated process such as hydrolysis, oxidation and precipitation, and continuous exposure to atmospheric CO_2 (g)), the $CaCO_3$ surrounding the

pore network dissolves and produces HCO_3^-} to neutralise the interfacial solution. We further highlight the role of degree of supersaturation with respect to $CaCO_3$ by observing the reduction in the pH in aerobic interfacial electrolytes surrounded by 10^{-3} mol/L $CaCO_3$. The pH decreases slowly from 8.4 to 8.05, in the same way and rate as in the case of [$CaCO_3$] = 10 mol/L until ≈ 36 hours (Figure S 6a, d). However, upon reaching undersaturation w.r.t. $CaCO_3$ at 36 hours, the acid neutralising capacity of the interfacial solution reduces further, and the pH decreases drastically by almost 1 unit over the next 36 hours (Figure S 5).

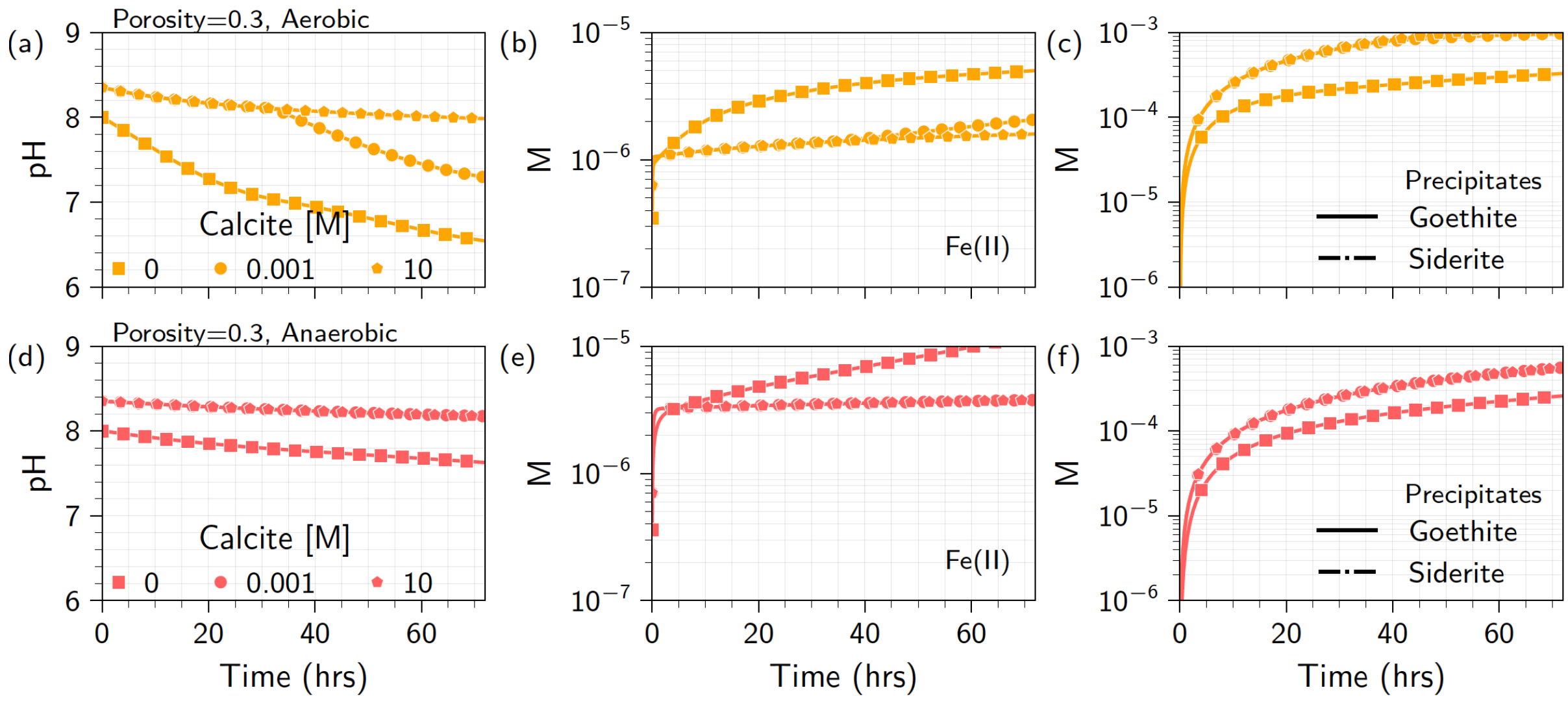

Figure S 6: (a)-(c) Evolution of pH, total ferrous complexes and precipitates respectively for aerobic conditions as a function of [$CaCO_3$]. (d)-(f) Evolution of pH, total ferrous complexes and precipitates respectively for anaerobic conditions as a function of [$CaCO_3$]. The results are shown for a region at a distance ~ 0.0025 mm from steel-porous medium interface. We consider 3 different starting concentrations (10 mol/l, 0.001 mol and 0 mol) of $CaCO_3$ and for a fixed fugacity of CO_2 at $10^{-3.5}$ atm. The one-dimensional porous medium has a porosity of 0.3.

The activity of dissolved oxygen, a very relevant parameter in electrochemical studies, has a significant influence on the chemistry of the interface. The decrease in the interfacial pH is more rapid and pronounced in aerobic environments (Figure 3i – main manuscript) than anaerobic conditions (Figure 3l – main manuscript). The higher rate of Fe^{II} oxidation (by roughly 2 orders of magnitude in aerobic conditions than anaerobic conditions over the pH of interest - Figure S 1c) in aerobic conditions is responsible for a higher concentration of Fe^{III} produced over a given time interval and therefore, the amount of goethite precipitated. Thus, the excess protons produced as a result of precipitation (Supplementary Note 5) are much

higher in aerobic conditions than anaerobic conditions and the decrease in the interfacial pH over time is higher (compare Figure 3i and Figure 3l – main manuscript).

The influence of oxygen on the interfacial chemistry is particularly evident in the absence of $CaCO_3$ and when the $[CaCO_3] = 10^{-3}$ mol/L. The lower rate of Fe^{II} oxidation under anaerobic conditions is responsible for a lower concentration of Fe^{III} produced over a given time interval and therefore, the amount of goethite precipitated. Thus, the excess protons produced as a result of precipitation are much lower in anaerobic conditions than aerobic conditions and the decrease in the interfacial pH over time is lower. Intuitively, we expect[8] the lower pH at the interface to facilitate an increase in the solubility of Fe^{II} and also a decrease in the Fe^{II} oxidation rate, resulting in increased $[Fe^{II}]$ over time. This argument is generally true and can explain the evolution of $[Fe^{II}]$ over time in individual scenarios for varying $[CaCO_3]$ and a particular oxygen concentration. However, upon comparing aerobic and anaerobic conditions, the $[Fe^{II}]$ is significantly higher in the latter case where the pH is relatively higher at all times. The primary reason for this is the slower rate of Fe^{II} oxidation under anaerobic conditions[1,23], which decreases the rate at which Fe^{II} is consumed.

5.3 *Solubility of Fe^{II} in the presence of $CaCO_3$*

The solubility of Fe^{II} is strongly influenced by the $[CaCO_3]$. As the concentration of $CaCO_3$ increases from 10^{-4} mol/L to 10 mol/L, the solubility of Fe^{II} increasingly depends on the aqueous iron-carbonate complexes, particularly in the near neutral pH range. Figure S 7 indicates the overall solubility of Fe^{II} and the contributions of each of the species formed in the Fe^{II} - $CaCO_3$-H_2O system as a function of the pH. It is worthwhile to note that in the presence of higher $[CaCO_3]$, the overall solubility of Fe^{II} is controlled by $FeCO_3$ (s), particularly at near neutral pH conditions ($6 < pH < 10$). However, $Fe(OH)_2$ (s) controls the solubility of Fe^{II} in alkaline conditions ($pH > 10$), irrespective of the $[CaCO_3]$.

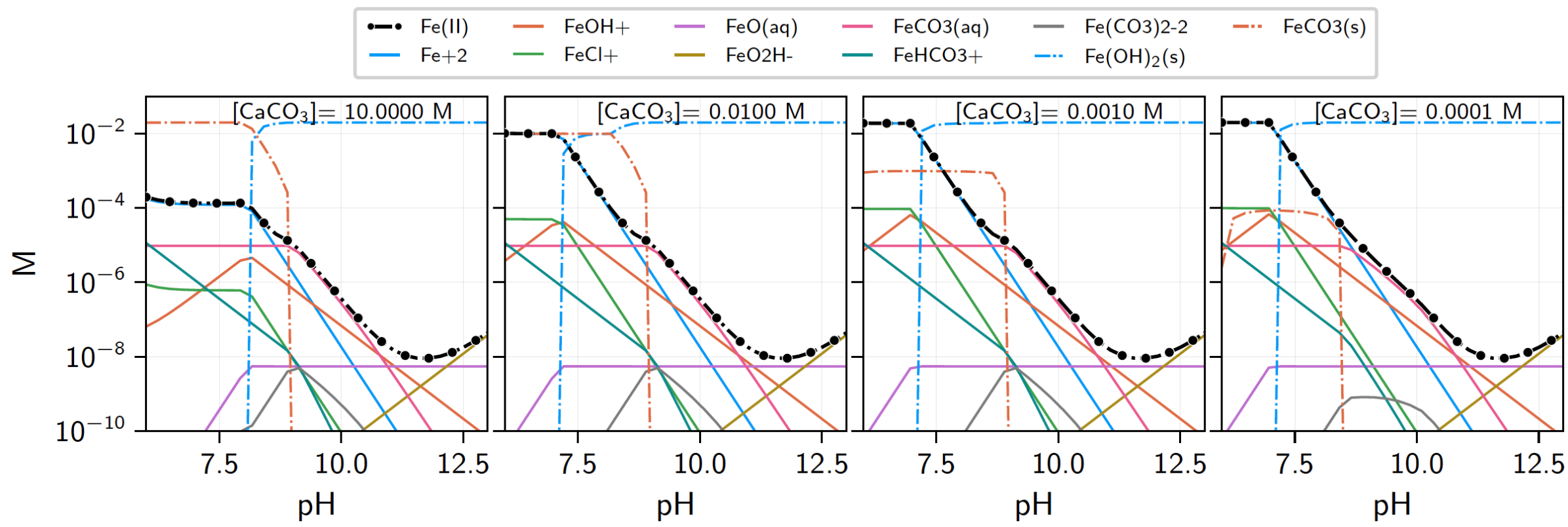

Figure S 7: Solubility of Fe^{II} under aerobic conditions in the presence of different concentrations of [$CaCO_3$]. A [Fe^{2+}] of 0.02 mol/L was used to compute the solubility of Fe^{II} species.

5.4 *Fe^{III} at the metal-porous medium interface and further away from the metal surface*

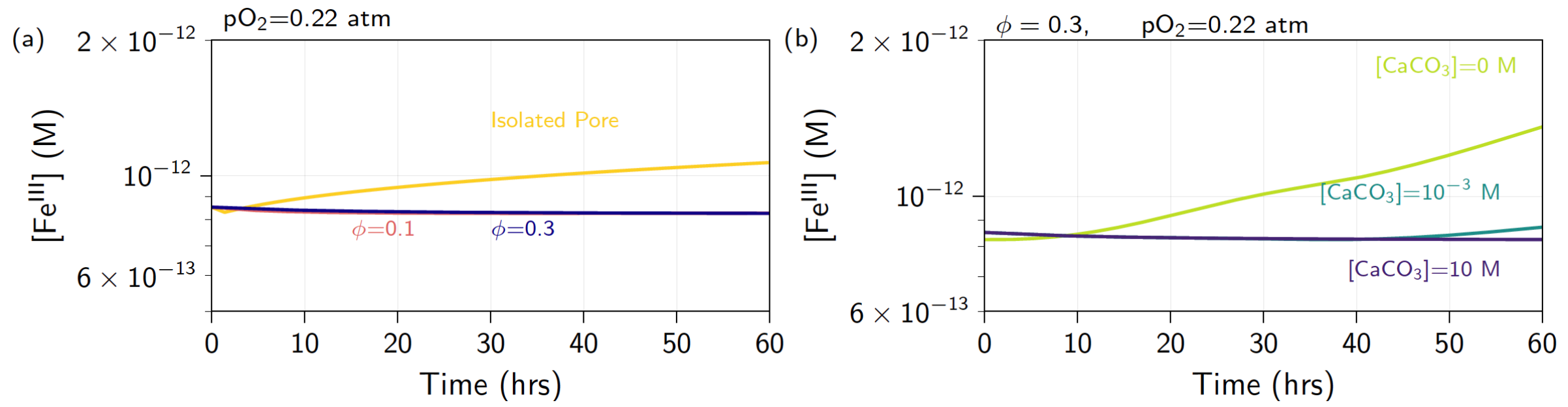

Figure S 8: Influence of (a) average porosity and (b) the [$CaCO_3$] (b) on the concentration of Fe^{III} over time at the metal-porous medium interface – corresponding to the simulation results shown in Figure 3 – main manuscript.

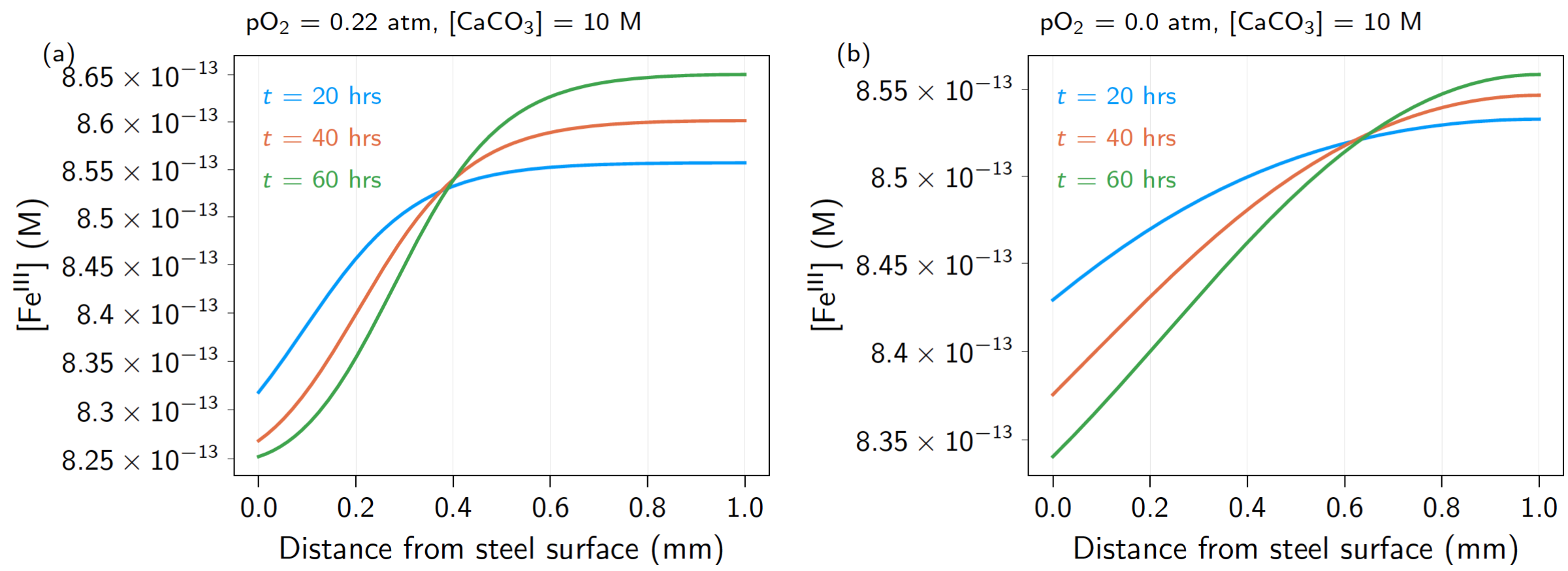

Figure S 9: Spatial and temporal distribution of $[Fe^{III}]$ in (a) aerobic and (b) anaerobic environments within the porous medium – corresponding to the simulation results shown in Figure 3 – main manuscript.

5.5 *Framework limitations*

First, we assume that Fe^{III} formed as a result of the oxidation of Fe^{II} ions instantly precipitate to its thermodynamically favoured solid state - goethite, and aqueous $[Fe^{III}]$ is dictated by the solubility limit of goethite (Figure S 8 and Figure S 9). It has been recently shown that thermodynamically less stable phases such as ferrihydrite may also precipitate[24] in the porous medium. It is certainly the case that thermodynamically less stable Fe^{III} (hydr)oxides (such as ferrihydrite) may transform to thermodynamically more stable states with time, which is kinetically controlled by the dissolution rate of the preceding solid phase[25]. As a first approximation, here, we do not consider the time-dependent transformation rate of different solid Fe^{III} (hydr)oxides. Furthermore, also the precipitation of corrosion products with iron in different oxidation states, such as magnetite (Fe_3O_4), has not been considered in this work but, will be addressed in detail in future studies (as highlighted in Section 4 – main manuscript).

Aqueous Fe^{III} is assumed to sorb on the surfaces of solid phases and cannot diffuse away. This assumption may not be entirely true. Therefore, in addition to the precipitation of Fe^{III} hydr(oxides), another sink term taking into account Fe^{III} sorption must be considered within the transient processes in our framework.

A second limitation of our study pertains to the reactive solid phase, $CaCO_3$, surrounding the electrolyte in the pore network. While we allow for the congruent dissolution of $CaCO_3$, we do not consider the kinetics of $CaCO_3$ dissolution as a function of the gradients in the pH, and

speciation of aqueous inorganic carbon at the surface of the dissolving $CaCO_3$ solid. To incorporate this, one would have to simulate processes occurring at the pore-scale within both the transient and equilibrium stages of this framework.

Thirdly, the precipitation of solid corrosion products, with a relatively higher molar volume compared to the bare metal within the pore network will change the local porosity and diffusivity of individual pores. It will also result in an increase in the expansive stresses exerted on the surrounding solids leading to the development of micro-cracks and subsequently altering the local pore structure. While, this is not the focus of this manuscript, one could incorporate temporal and spatial changes in the pore structure into the framework.

Finally, in the relevant example of steel corrosion in carbonated concrete and to illustrate the applicability of our reactive transport framework, we did not alter the Fe^0 corrosion rate over time and instead, used a constant flux of Fe^{II} and correspondingly, $[OH^-]$. It has been established that the corrosion rate, under practical exposure conditions depends on the physical and chemical characteristics (fraction of saturated pores at the steel/porous media interface, the concentration of iron ions at the interface, diffusion of iron ions away from the corroding interface - dependent on porosity and connectivity) of the steel/porous media interface[26]. In future studies we aim to incorporate this by changing the input flux accordingly in our framework.

References

(1) Mundra, S.; Tits, J.; Wieland, E.; Angst, U. M. Aerobic and Anaerobic Oxidation of Ferrous Ions in Near-Neutral Solutions. *Chemosphere* **2023**, *335*, 138955. https://doi.org/10.1016/j.chemosphere.2023.138955.

(2) Morgan, J. J. Kinetics of Reaction between O2 and Mn(II) Species in Aqueous Solutions. *Geochim Cosmochim Acta* **2005**, *69* (1), 35–48. https://doi.org/10.1016/j.gca.2004.06.013.

(3) Klewicki, K. J. The Kinetics of Redox Reactions of Mn(II) and Mn(III) in Aqueous Systems: Homogeneous Autoxidation of Mn(II) and the Formation and Disappearance of Mn(III) Complexes, California Institute of Technology, Pasadena, 1996.

(4) Johnson, K. S.; Coale, K. H.; Berelson, W. M.; Gordon, R. M. On the Formation of the Manganese Maximum in the Oxygen Minimum. *Geochim Cosmochim Acta* **1996**, *60* (8), 1291–1299. https://doi.org/10.1016/0016-7037(96)00005-1.

(5) Lemire, R. J.; Berner, U.; Musikas, C.; Palmer, D. A.; Taylor, P.; Tochiyama, O. *Chemical Thermodynamics of Iron: Part 1*; Nuclear Energy Agency of the OECD, 2013; Vol. 13a.

(6) Robie, R. A.; Hemingway, B. S. *Thermodynamic Properties of Minerals and Related Substances at 298.15 K and 1 Bar (10^5 Pascals) Pressure and at Higher Temperatures*; U.S Geological Survey Bulletin 2131: Washington D.C., 1995.

(7) Brown, P. L.; Ekberg, C. *Hydrolysis of Metal Ions*; Wiley-VCH, 2016; Vol. 1. https://doi.org/10.1002/9783527656189.ch11.

(8) Furcas, F. E.; Lothenbach, B.; Isgor, O. B.; Mundra, S.; Zhang, Z.; Angst, U. M. Solubility and Speciation of Iron in Cementitious Systems. *Cem Concr Res* **2022**, *151*, 106620.

(9) Hummel, W.; Thoenen, T. *The PSI Chemical Thermodynamic Database 2020*; Nagra: Wettingen, 2023.

(10) Lemire, R. J.; Palmer, D. A.; Costa, D.; Martinez, J. *Chemical Thermodynamics of Iron: Part 2*; Nuclear Energy Agency of the OECD, 2020; Vol. 13b.

(11) Dilnesa, B. Z.; Lothenbach, B.; Le Saoût, G.; Renaudin, G.; Mesbah, A.; Filinchuk, Y.; Wichser, A.; Wieland, E. Iron in Carbonate Containing AFm Phases. *Cem Concr Res* **2011**, *41* (3), 311–323. https://doi.org/10.1016/j.cemconres.2010.11.017.

(12) Lothenbach, B.; Kulik, D. A.; Matschei, T.; Balonis, M.; Baquerizo, L.; Dilnesa, B.; Miron, G. D.; Myers, R. J. Cemdata18: A Chemical Thermodynamic Database for Hydrated Portland Cements and Alkali-Activated Materials. *Cem Concr Res* **2019**, *115*, 472–506.

(13) Hummel, W.; Berner, U.; Curti, E.; Pearson, F. J.; Thoenen, T. *Nagra/PSI Chemical Thermodynamic Data Base 01/01*; Universal Publishers: Parkland, Florida, 2002.

(14) von Greve-Dierfeld, S.; Lothenbach, B.; Vollpracht, A.; Wu, B.; Huet, B.; Andrade, C.; Medina, C.; Thiel, C.; Gruyaert, E.; Vanoutrive, H.; Saéz del Bosque, I. F.; Ignjatovic, I.; Elsen, J.; Provis, J. L.; Scrivener, K.; Thienel, K. C.; Sideris, K.; Zajac, M.; Alderete, N.; Cizer, Ö.; Van den Heede, P.; Hooton, R. D.; Kamali-Bernard, S.; Bernal, S. A.; Zhao, Z.; Shi, Z.; De Belie, N. Understanding the Carbonation of Concrete with Supplementary Cementitious Materials: A Critical Review by RILEM TC 281-CCC. *Mater Struct* **2020**, *53* (6). https://doi.org/10.1617/s11527-020-01558-w.

(15) Angst, U.; Moro, F.; Geiker, M.; Kessler, S.; Beushausen, H.; Andrade, C.; Lahdensivu, J.; Köliö, A.; Imamoto, K. I.; von Greve-Dierfeld, S.; Serdar, M. Corrosion of Steel in Carbonated Concrete: Mechanisms, Practical Experience, and Research Priorities – A Critical Review by RILEM TC 281-CCC. *RILEM Technical Letters* **2020**, *5*, 85–100. https://doi.org/10.21809/rilemtechlett.2020.127.

(16) Stefanoni, M.; Angst, U.; Elsener, B. The Mechanism Controlling Corrosion of Steel in Carbonated Cementitious Materials in Wetting and Drying Exposure. *Cem Concr Compos* **2020**, *113*, 103717.

(17) Sulpis, O.; Boudreau, B. P.; Mucci, A.; Jenkins, C.; Trossman, D. S.; Arbic, B. K.; Key, R. M. Current CaCO3 Dissolution at the Seafloor Caused by Anthropogenic CO2. *Proc Natl Acad Sci U S A* **2018**, *115* (46), 11700–11705. https://doi.org/10.1073/pnas.1804250115.

(18) Schnoor, J. L.; Stumm, W. The Role of Chemical Weathering in the Neutralization of Acidic Deposition. *Swiss Journal of Hydrology* **1986**, *48* (2), 171–195. https://doi.org/10.1007/BF02560197.

(19) Sulpis, O.; Jeansson, E.; Dinauer, A.; Lauvset, S. K.; Middelburg, J. J. Calcium Carbonate Dissolution Patterns in the Ocean. *Nat Geosci* **2021**, *14* (6), 423–428. https://doi.org/10.1038/s41561-021-00743-y.

(20) Feely, R. A.; Sabine, C. L.; Lee, K.; Berelson, W.; Kleypas, J.; Fabry, V. J.; Millero, F. J. Impact of Anthropogenic CO2 on CaCO3 System in the Oceans. *Science (1979)* **2004**, *305*, 362–366. https://doi.org/10.2134/jae1985.0003.

(21) Morse, J. W.; Arvidson, R. S.; Lüttge, A. Calcium Carbonate Formation and Dissolution. *Chem Rev* **2007**, *107* (2), 342–381.

(22) Morse, J. W.; Andersson, A. J.; Mackenzie, F. T. Initial Responses of Carbonate-Rich Shelf Sediments to Rising Atmospheric PCO2 and "Ocean Acidification": Role of High Mg-Calcites. *Geochim Cosmochim Acta* **2006**, *70* (23 SPEC. ISS.), 5814–5830. https://doi.org/10.1016/j.gca.2006.08.017.

(23) Stumm, W.; Morgan, J. J. *Aquatic Chemistry: Chemical Equilibria and Rates in Natural Waters*, Third Edition.; John Wiley & Sons, Ed.; 1996.

(24) Albert, C. C.; Mundra, S.; Ferreira Sanchez, D.; Furcas, F. E.; Rajyaguru, A. D.; Isgor, O. B.; Grolimund, D.; Angst, U. M. Microscale Chemical Imaging to Characterize and Quantify Corrosion Processes at the Metal-Electrolyte Interface. *Npj Mater Degrad* **2024**, *8* (1), 116.

(25) Furcas, F. E.; Lothenbach, B.; Mundra, S.; Borca, C. N.; Albert, C. C.; Isgor, O. B.; Huthwelker, T.; Angst, U. M. Transformation of 2-Line Ferrihydrite to Goethite at Alkaline PH. *Environ Sci Technol* **2023**, *57* (42), 16097–16108. https://doi.org/10.1021/acs.est.3c05260.

(26) Stefanoni, M.; Angst, U. M.; Elsener, B. Kinetics of Electrochemical Dissolution of Metals in Porous Media. *Nat Mater* **2019**, *18*, 942–947.
